# Overview of the Design and Application of Photothermal Immunoassays

**DOI:** 10.3390/s24196458

**Published:** 2024-10-06

**Authors:** Fengli Gao, Yike Wu, Cui Gan, Yupeng Hou, Dehua Deng, Xinyao Yi

**Affiliations:** 1 Henan Province Key Laboratory of New Opto-Electronic Functional Materials, College of Chemistry and Chemical Engineering, Anyang 455000, China; flgao@aynu.edu.cn (F.G.); 19711083859@163.com (Y.W.); m17796544225@163.com (C.G.); 16692208021@163.com (Y.H.); 2 College of Chemistry and Chemical Engineering, Central South University, Changsha 410083, China

**Keywords:** immunoassays, photothermal, nanozymes, noble metal nanomaterials

## Abstract

Developing powerful immunoassays for sensitive and real-time detection of targets has always been a challenging task. Due to their advantages of direct readout, controllable sensing, and low background interference, photothermal immunoassays have become a type of new technology that can be used for various applications such as disease diagnosis, environmental monitoring, and food safety. By modification with antibodies, photothermal materials can induce temperature changes by converting light energy into heat, thereby reporting specific target recognition events. This article reviews the design and application of photothermal immunoassays based on different photothermal materials, including noble metal nanomaterials, carbon-based nanomaterials, two-dimensional nanomaterials, metal oxide and sulfide nanomaterials, Prussian blue nanoparticles, small organic molecules, polymers, etc. It pays special attention to the role of photothermal materials and the working principle of various immunoassays. Additionally, the challenges and prospects for future development of photothermal immunoassays are briefly discussed.

## 1. Introduction

Immunoassays based on the highly specific antigen-antibody interactions are considered as the gold standards for sensitive detection of various analytes due to their advantages of high sensitivity, excellent universality, and powerful on-site monitoring ability [1,2]. They have been widely used in the fields of clinical diagnosis, environmental assessment, and food safety [3,4,5]. Enzyme-linked immunosorbent assay (ELISA), as one of the most commonly used methods, has been widely applied in target detection and quantification [6]. In order to meet the requirements of ultrasensitive detection of low abundance in complex samples, different techniques have been combined with ELISA [7]. According to the type of output signal, immunoassay methods can be divided into colorimetric, fluorescent, chemiluminescence, surface-enhanced Raman spectroscopy, electrochemical, electrochemiluminescence, and photoelectrochemical immunosensors [8,9,10]. Despite the tremendous achievements and rapid development of these immunoassay technologies [11], they still face inherent limitations, such as expensive instruments, specialized operators, and complex operating procedures. Alternatively, point-of-care testing (POCT) immunoassay has been popularly developed in recent years with cost-effectiveness, portability, simplicity, specificity, and user friendliness, making it highly suitable for real-time analysis in resource-limited regions [12,13,14]. For example, colorimetric lateral flow immunoassay (LFIA), a versatile and convenient POCT method due to its well-known merits of low cost and user friendliness, has been commercially used for pregnancy and COVID-19 tests [15,16]. Nonetheless, the color change is always confronted with poor color resolution for the bare eye-detectable readout, and the colorimetric LFIA only provides qualitative or semiquantitative results. Therefore, it is of great importance to develop simple and objective POCT immunoassay methods for quantitative analysis of biomolecules.

In a typical POCT system, the transducer can transform a molecular recognition event into a measured or observed signal, playing a critical role in target measurement [17]. To break the inherent limitation of colorimetric POCT devices, various novel strategies have been integrated with LFIA to provide supplementary signals for quantitative analysis. For example, a series of different minimal and simple devices have been coupled with competitive or sandwich immunoassays using certain functional labels, such as thermometers [18,19], glucose meters [20,21], electronic balances [22,23], and pressure meters [24]. Temperature, such as room temperature, body temperature, and water temperature, is a common physical parameter in daily life. A large number of photothermal immunoassays have been reported by using thermometers as the readers due to their excellent advantages of straightforward readout, more controllable sensing, low background interference, and less susceptibility to optical drift [25,26]. Generally, there are two ways for integrating photothermal materials into photothermal immunoassays, and the temperature change is correlated with target concentration, which can be easily measured by conventional thermometers, digital thermometers, or thermal imagers without the use of advanced analytical instruments. One way is to modify photothermal reagents with antibodies by physical adsorption and covalent coupling and use them as photothermal labels in the detection platform. After the specific antigen-antibody interaction, the amount of photothermal agents in the detection solution is linearly related to the increase of solution temperature under laser irradiation. Another method involves the use of photothermal agents as the substrates in immunoassay. The photothermal response of the system can be regulated by in situ aggregation, production, consumption, or growth/etching of photothermal agents triggered by the biomolecular reducing or oxidizing reagents.

In recent years, a number of new photothermal immunoassay platforms have emerged and developed. A few excellent reviews have been reported to address the development of photothermal biosensors [27,28,29,30,31,32]. For example, Wei et al. summarized the progress of the newly developed photothermometric assays based on various photothermal materials [31]. Ouyang et al. reviewed the recent progress in photothermal nanomaterials-based determination of circulating tumor cells [33]. However, there is no special issue to systematically summarize the design and application of various photothermal immunoassays. With the development of analytical techniques and materials engineering, many new progresses have been achieved in photothermal immunoassays, and an updated and comprehensive overview of this field is desired. In this review, we focus on the latest advances in photothermal immunoassays by summarizing different sensing strategies with various photothermal agents and materials. The challenges and perspectives of photothermal immunoassays for future development are also briefly discussed.

## 2. Photothermal Immunoassays with Different Materials

Developing photothermal materials with high light-to-heat conversion efficiency is an important foundation for improving the detection performance of immunoassays. In recent years, a series of photothermal materials have been explored for bioassays and imaging, including noble metal nanomaterials [34,35], carbon-based nanostructures [36], two-dimensional (2D) nanomaterials [37], metal oxide and sulfide nanomaterials [38], Prussian blue (PB) NPs [39,40], small organic molecules, and polymers [41,42]. In addition, most of the photothermal materials are colored with the absorbance spectrum ranging from visible to NIR regions, thus providing a basis for colorimetric/photothermal dual-signal assays. In this section, we systematically discussed the design and application of photothermal immunoassays according to the difference in the type of used materials.

### 2.1. Noble Metal Nanomaterials

Noble metal nanomaterials, especially gold nanoparticles (AuNPs) and silver nanoparticles (AgNPs), exhibit unique local surface plasmon resonance (LSPR) absorption characteristics. The frequency and intensity of LSPR bands are highly correlated with the size, shape/morphology, metal composition, dielectric environment, and distance between adjacent nanoparticles [43,44]. When the laser wavelength is matched with the LSPR wavelength of noble metal nanostructures, free electrons on the metal surface can be collectively excited to induce strong coherent oscillation. A large amount of laser energy is absorbed and converted into heat, leading to an increase in environmental temperature. Therefore, the photothermal effect of noble metal nanostructures, including nanoparticles, nanorods, nanostars, and nanobipyramids, has been thoroughly explored for widespread application in photothermal immunoassays [45,46]. According to previous reports, it is a feasible strategy to modulate the photothermal effect by adjusting the LSPR absorption wavelength of plasmonic nanomaterials, such as the dispersion and aggregation of AuNPs and the in situ growth or etching of nanostructures. In this section, photothermal immunoassays with noble metal nanomaterials as signal labels and substrates are individually discussed.

#### 2.1.1. Signal Labels

**Figure 1:**
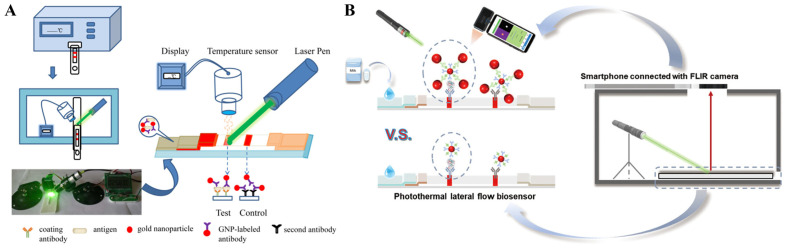
(**A**) Schematic illustration of a rapid and quantitative LFIA using AuNPs as photothermal agents [51]. Copyright 2019 Elsevier. (**B**) Schematic illustration of a smartphone-integrated photothermal LFIA using dual AuNPs conjugates as labels [53]. Copyright 2024 American Chemical Society.

It has been demonstrated that sphere AuNPs can be used as photothermal labels to improve the detection sensitivity of immunoassays compared to the visual detection methods (Table 1) [47,48,49,50]. For example, Zhang et al. reported a rapid and quantitative LFIA method based on the photothermal effect of AuNPs (Figure 1A) [51]. The coating antibody and second antibody were immobilized on the nitrocellulose membrane as the test line and control line, respectively. Upon laser irradiation at 532 nm with a laser pen, the temperature change of the test line was monitored by a temperature sensor. The detection limit of LFIA (5.5 × 10^2^ CFU/mL) was at least one order of magnitude lower than the AuNPs-LFIA visual detection (9.92 × 10^3^ CFU/mL). Liu et al. constructed a four-channel photothermal plate reader for high-throughput detection of C-reaction protein (CRP) with glutathione-coated AuNPs as the photothermal labels [52]. However, the AuNPs-based methods rely on laser irradiation with a wavelength of 520 nm coinciding with the LSPR absorption peak, thus showing relatively high background signal and low signal-to-noise ratio. To enhance the photothermal effect of AuNPs-based nanoprobes, Huang et al. developed a smartphone-integrated photothermal LFIA platform by using dual AuNPs conjugates as the signal labels [53]. As shown in Figure 1B, the conjugates were prepared by coupling 15 nm AuNPs with 40 nm AuNPs, and their LSPR absorption band was much stronger than that of 15 nm AuNPs, thereby improving the photothermal effect. The photothermal conversion efficiency of dual AuNPs conjugates, 40 nm AuNPs, and 15 nm AuNPs was found to be 24.0%, 9.30%, and 7.09%, respectively. The detection limits for colorimetric and photothermal immunoassays of staphylococcus enterotoxin A (SEA) were 0.091 ng/mL and 0.0038 ng/mL, respectively, which were 10.7-fold and 255.3-fold lower than those of 15 nm AuNPs.

**Figure 2:**
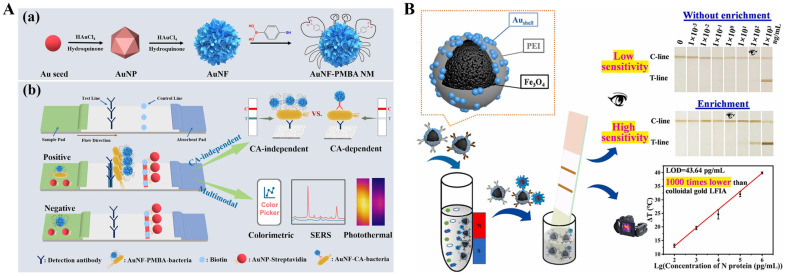
(**A**) Schematic illustration of this novel MCI-LFIA biosensor based on AuNF-PMBA NMs for diagnosis of bacterial UTI. (**a**): Preparation of AuNF–PMBA NMs. (**b**): Principle of MCI-LFIA for bacterial detection [63]. Copyright 2023 Elsevier. (**B**) Schematic illustration of a photothermal and colorimetric LFIA for dual-mode determination of SARS-CoV-2 N protein using Au nanoshell-coated Fe_3_O_4_ nanoclusters [67]. Copyright 2023 Elsevier.

The photothermal effect of AuNPs can be improved by changing their composition, structure, shape, and other factors, eventually affecting the LSPR band and enhancing the photothermal effect [54,55]. For instance, anisotropic gold nanostructures were preferred to develop photothermal immunoassays because they exhibit extended LSPR absorption peaks from visible to NIR regions [56,57]. Gold nanorods (AuNRs) exhibit tunable LSPR absorption bands from UV-visible to NIR regions by readily controlling the length-to-diameter ratio, and their photothermal conversion efficiency varies from 50% to 95% [58,59]. Therefore, AuNRs have been successfully used as photothermal reagents for the detection of metal ions and glucose and the immunoassays of different targets [60,61,62]. For instance, Liu et al. proposed a colorimetric and photothermal LFIA platform for the detection of SARS-CoV-2 using NIR-responsive AuNPs as photothermal labels [62]. The LSPR peak of AuNRs was tuned to match the wavelength of NIR emission lasers (808 nm), achieving maximum photothermal conversion efficiency and best photothermal detection performance. In addition, gold nanoflowers (AuNFs) with sharp edges and multiple branching angles are good substrates for SERS nanotags and excellent colorimetric/photothermal labels because of their dark blue-green color and wide absorption bands from 500 nm to 900 nm. Wu et al. reported a multimodal LFIA method for the detection of bacterial urinary tract infections without the use of a capture antibody based on *p*-mercaptophenylboronic acid (PMBA)-modified AuNFs (Figure 2A) [63]. PMBA tags on the surface of AuNFs could specifically react with the *cis*-diol species in the peptidoglycan on the bacteria surface through the formation of cyclic *cis*-diol esters. Meanwhile, PMBA serving as a Raman reporter exhibited a characteristic peak at 1066 cm^−1^. Thus, PMBA-AuNFs could be used to effectively capture both *Gram-positive* and *Gram-negative* bacteria and generate colorimetric-Raman-photothermal triple signals. Magnetic materials can facilitate the separation and enrichment of targets in complex biological samples under an extra magnetic field. The combination of magnetic materials and AuNPs can improve the sensitivity and anti-interference ability [64,65,66]. Li et al. reported a photothermal and colorimetric LFIA platform for dual-mode determination of SARS-CoV-2 nucleocapsid protein (N protein) by using Au nanoshell-coated Fe_3_O_4_ nanoclusters (MagAu_shell_) (Figure 2B) [67]. PEI molecules were used as adhesives for assembling Fe_3_O_4_ nanoclusters and Au nanoshells. The formed MagAu_shell_ exhibited comprehensive magnetic and photothermal properties and good stability. The photothermal conversion efficiency of Au nanoshells, MagAu_shell_ NPs, and Fe_3_O_4_ nanoclusters was found to be 64.71%, 26.90%, and 10.46%, respectively. There was no significant effect on the highest temperature attainable and the time cost by heating and cooling MagAu_shell_ repeatedly for several times. The capture of SARS-CoV-2 N protein allowed for the attachment of antibody-modified MagAu_shell_, thereby resulting in a brown stripe on the T-line and generating an obvious photothermal signal under infrared laser irradiation at 808 nm.

**Figure 3:**
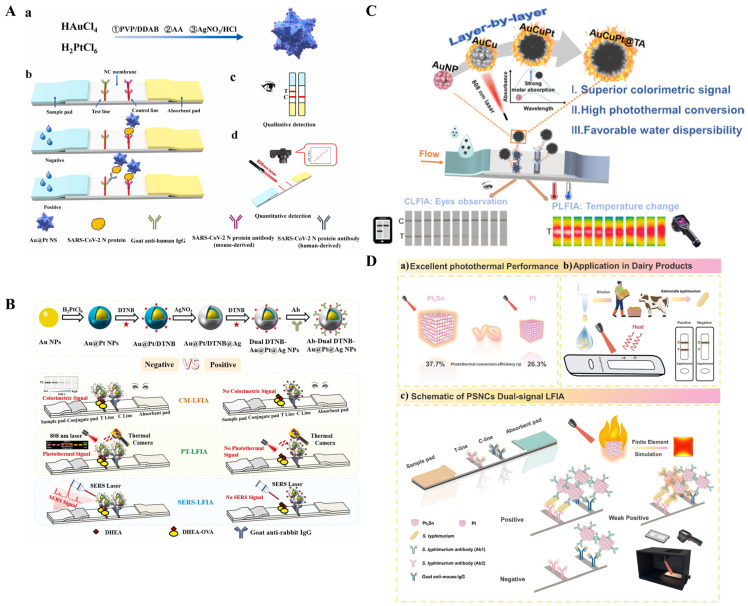
(**A**) Schematic illustration of an Au@Pt nanostars-based dual-signal LFIA for colorimetric and photothermal detection of SARS-CoV-2 nucleocapsid antibody [71]. Copyright 2024 Elsevier. (**B**) Schematic illustration of a multimodal LFIA for DHEA detection using multifunctional Au@Pt@Ag NPs with color-photothermal-Raman properties [73]. Copyright 2022 Elsevier. (**C**) Schematic illustration of a colorimetric and photothermal LFIA based on spark-type AuCuPt alloy for the detection of estriol [75]. Copyright 2024 American Chemical Society. (**D**) Schematic illustration of (**a**) photothermal performance comparison of bimetallic nanocuboid Pt_3_Sn and Pt NPs; (**b**) detection of *S. typhimurium* in dairy product samples; (**c**) principle for the detection of *S. typhimurium* Using Pt_3_Sn-based LFIA [77]. Copyright 2024 American Chemical Society.

In contrast to single-element metallic nanoparticles, bimetallic nanomaterials, such as core-shell Au@Ag NPs [68], Ag–Au urchin-like hollow nanospheres [69], and Au nanoclusters@PtOs nanoclusters [70], have been proven to possess stronger photothermal conversion efficiency under NIR laser irradiation due to their higher absorption capacity in the visible region. Meanwhile, their absorption coefficients and deeper colors can greatly enhance the sensitivity of colorimetric immunoassays, allowing for qualitative detection of different targets in multiple modes. Zhu et al. reported an Au@Pt nanostars-based dual-signal LFIA method for colorimetric and photothermal detection of SARS-CoV-2 nucleocapsid antibody (Figure 3A) [71]. In this study, Au@Pt stellate nanostars were prepared via a one-pot method and then modified with the N protein of SARS-CoV-2. Based on the specific antigen-antibody interactions, deeper colors were observed for positive samples in both T and C lines, but only the C line showed color in negative samples. Under irradiation with an 808 nm laser, the temperature change in the T-line was recorded by a handheld thermal imager. The detection limit for the photothermal mode was 24.91 pg/mL, which is about 40-fold lower than that of the colorimetric detection. In addition, Pt NPs and their hybrids with other materials have been proven to possess excellent intrinsic peroxidase-like activity. Jiao et al. reported an Au@Pt nanodendrites-enhanced multimodal ELISA for cardiac troponin I (cTnI) detection [72]. In this work, Au@Pt nanodendrites with good photothermal effect generated an enhanced temperature under 808 nm laser irradiation, which could be measured by a hand-held thermometer. Meanwhile, the Au@Pt nanodendrites with intrinsic peroxidase-like activity could catalyze the oxidization of *o*-phenylenediamine (OPD) into 2,3-diaminophenazine (OPDox) with a yellow color change. The produced OPDox showed a maximum emission peak at 580 nm, which can quench the fluorescence of carbon dots (CDs) at 443 nm, realizing the ratiometric fluorescence detection of cTnI. Moreover, the internal and external “hotspot” effect of core-shell nanoparticles can produce strong SERS signals with excellent reproducibility. Yang et al. developed a multimodal LFIA platform for dehydroepiandrosterone (DHEA) detection based on multifunctional Au@Pt@Ag NPs with color-photothermal-Raman triple capabilities [73]. As displayed in Figure 3B, core-shell-shell Au@Pt@Ag NPs modified with dual-layer Raman reporter molecules (5,5′-dithiobis-(2-nitrobenzoic acid, DTNB)) were used as multifunctional nanotags with triple properties. Au@Pt@Ag NPs showed a strong absorption in the UV-Vis and even NIR regions, providing stable and extensive photothermal signals in LFIA. The photothermal conversion efficiency of Au@Pt NPs was 46.2%, and that of Au@Pt@Ag NPs with an average of 66.4 nm, 76.1 nm, 91.8 nm, and 153.0 nm was found to be 46.5%, 51.8%, 46.5%, and 44.3%, respectively. The double layers of DTNB inside and outside of Au@Pt@Ag NPs produced high Raman signals. The characteristic brown-gray color can be directly observed by naked eyes for colorimetric detection of DHEA.

The reasonable combination of precious metals in alloy nanoparticles can greatly improve the molar extinction coefficient and photothermal conversion efficiency [74]. Recently, Wu et al. reported a colorimetric and photothermal LFIA platform by using spark-type AuCuPt alloys for the detection of estriol (Figure 3C) [75]. In this work, a trimetallic AuCuPt alloy with a rational design of metal components was prepared via a one-step reduction method and then modified with tannic acid (TA) for high biocompatibility. The combination of Au and Cu led to excellent photothermal effect. The molar extinction coefficient and photothermal conversion efficiency reached 2.38 × 10^12^ M^−1^cm^−1^ and 48.5%, respectively, due to the introduction of Pt. Based on the unique optical properties of AuCuPt alloys, the detection limits for the dual-signal LFIA platform were 0.033 ng/mL (colorimetric) and 0.021 ng/mL (colorimetric). Similarly, Yin et al. prepared AgPdCu alloy hollow nanospheres with high photothermal conversion efficiency for the detection of Albendazole in LFIA [76]. In addition to the introduction of noble metals, Shi et al. successfully synthesized doping engineering-powered bimetallic nanocuboid Pt_3_Sn nanostructures and used them to develop a dual-signal LFIA platform for *S. typhimurium* detection (Figure 3D) [77]. In this study, the antibody-modified Pt_3_Sn nanostructures were used to capture *S. typhimurium* and then attach onto the T-line. The captured nanostructures exhibited bright black color for colorimetric detection and showed excellent photothermal conversion efficiency for photothermal analysis. The detection limits for the colorimetric and photothermal immunoassays were 10^3^ cfu/mL and 10^2^ cfu/mL, respectively, which were 100 times lower than the traditional AuNPs-based methods.

**Table 1:** Performances of photothermal immunoassays with noble metal nanomaterials as signal labels.

Signal Label	Target	Linear Range	Detection Limit	Ref.
AuNPs	*E. coli*	2 × 10^4^–2 × 10^7^ CFU/mL	1.95 × 10^4^ CFU/mL	[48]
AuNPs	CEA	0.67–7.27 ng/mL	30 pg/mL	[49]
AuNPs	AFB1	0.5–500 ng/mL	0.27 ng/mL	[50]
AuNPs	*S. T*	1.2 × 10^6^–3.4 × 10^7^	1.2 × 10^6^ CFU/mL	[51]
AuNPs	CRP	10–500 μg/mL	0.52 ng/mL	[52]
AuNPs	SEA	0.005–10 ng/mL	3.8 pg/mL	[53]
Au_shell_ NPs	IL-6	1–1000 ng/mL	0.3 ng/mL	[55]
AuNPs	N protein	Not reported	96 pg/mL	[62]
AuNF-PMBA	*E. coli*	10^2^–10^7^ CFU/mL	100 CFU/mL	[63]
Fe_3_O_4_@Au	IL-6	5 × 10^−5^–1 ng/mL	21.3 fg/mL	[64]
Au-Fe_3_O_4_	Lp-PLA_2_	0.01–100 ng/mL	8.6 ng/mL	[65]
Au-Fe_3_O_4_	*S. T*	5 × 10^6^–1 × 10^8^ CFU/mL	5 × 10^4^ CFU/mL	[66]
MagAu_shell_	N protein	100–1000 pg/mL	43.64 pg/mL	[67]
Au^4-ATP^@Ag NPs	IAV	Not reported	15.63 pg/mL	[68]
BUHNPs	*E. coli*	0–5 × 10^6^ CFU/mL	550 CFU/mL	[69]
Au@PtOs	Exosome	9.6 × 10^2^–1.2 × 10^5^ exosomes/μL	460 exosomes/μL	[70]
Au@Pt NSs	N protein antibody	10^−8^–10^−3^ mg/mL	24.91 pg/mL	[71]
Au@Pt	cTnI	0.5–5 ng/mL	0.34 ng/mL	[72]
Au@Pt@Ag	DHEA	1–1000 ng/mL	0.42 ng/mL	[73]
Ap@PtNp	*E. coli*	10^1^–10^7^ CFU/mL	14 CFU/mL	[74]
AuCuPt@TA	Estriol	0.01–0.75 ng/mL	21 pg/mL	[75]
AgPd-CuHNs	ABZ	0.279–100 ng/mL	18 pg/mL	[76]
Pt_3_Sn	*S. T*	10^2^–10^7^ CFU/mL	10^0^ CFU/mL	[77]

Abbreviation: AuNPs, gold nanoparticles; *E. coli O157:H7*, *Escherichia coli O157:H7*; CEA, carcinoembryonic antigen; AFB1, aflatoxin B1; *S. T*, *Salmonella typhimurium*; CRP, C-reactive protein; SEA, staphylococcus enterotoxin A; Au_shell_, Au nanoshell; IL-6, Interleukin 6; N protein, SARS-CoV-2 nucleocapsid protein; AuNF-PMBA, *p*-mercaptophenylboronic acid-modified gold nanoflowers; Au-Fe_3_O_4_, Au-coated magnetic Fe_3_O_4_ core-shell nanohybrids; Lp-PLA_2_, lipoprotein-associated phospholipase A_2_; IAV, influenza A virus; Au^4-ATP^@Ag NPs, gold-core-silver-shell bimetallic nanoparticles; BUHNPs, bimetallic Ag–Au urchin-like hollow nanospheres; AuNSs, gold nanostars; cTnI, cardiac troponin I; DHEA, dehydroepiandrosterone; Ap@PtNp, platinum nanoparticle organic-inorganic composites; AuCuPt@TA, AuCuPt nanoflowers modified with tannic acid; AgPd-CuHNs, AgPdCu hollow nanospheres; ABZ, albendazole.

#### 2.1.2. Photothermal Substrates

**Figure 4:**
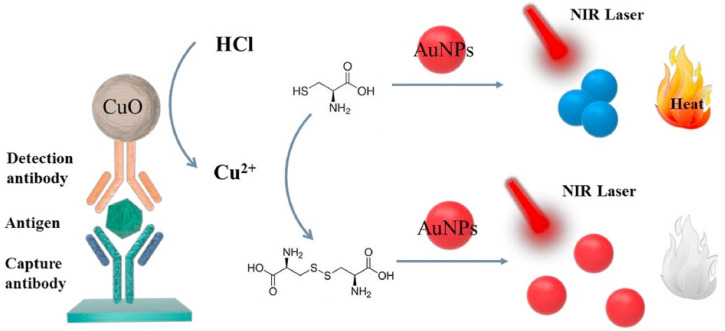
Schematic illustration of a photothermal immunoassay for CEA detection based on Cu^2+^-catalyzed consumption of Cys and Cys-induced aggregation of AuNPs [85]. Copyright 2021 Elsevier.

Traditional enzyme-labeled immunoassays can be integrated with plasmonic nanoparticles-based photothermal platforms by adjusting the dispersed state of AuNPs and precisely regulating the morphology/shape and size of plasmonic nanostructures to modulate the characteristic LSPR bands [78]. The target-triggered aggregation of AuNPs can lead to a red shift of the LSPR absorption band and a significant NIR-responsive photothermal change [79]. Based on the different photothermal effects of aggregated and dispersed AuNPs, various targets have been determined by thermometers, such as adenosine [80], p53 DNA [81], carbendazim [82], ochratoxin A (OTA) [83], and *Mycobacterium tuberculosis* DNA [84]. Tao et al. developed a photothermal immunoassay platform for carcinoembryonic antigen (CEA) detection based on Cu^2+^-catalyzed consumption of cysteine (Cys) and Cys-induced aggregation of AuNPs [85]. As illustrated in Figure 4, Cys induced the aggregation of AuNPs due to the formation of Au-S bonds. The dispersed red AuNPs with weak photothermal effect in the NIR region were assembled into blue aggregates with strong photothermal effect, producing a remarkable increase in solution temperature. CuO NPs loaded with antibodies could release a large number of Cu^2+^ ions in the presence of HCl. Then, the released Cu^2+^ catalyzed the oxidation of Cys into cystine and inhibited the Cys-induced aggregation of AuNPs. The introduction of Cu^2+^ caused a weak photothermal response of the system, which can be readily recorded by a common thermometer. The photothermal immunoassay achieved a wide detection range from 3 to 48 ng/mL with a detection limit of 1.3 ng/mL.

**Figure 5:**
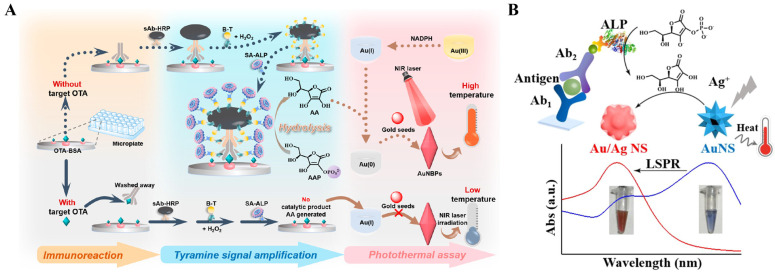
(**A**) Schematic illustration of a photothermal immunoassay for OTA detection based on the in situ growth of AuNBPs [91]. Copyright 2023 American Chemical Society. (**B**) Schematic illustration of a colorimetric and photothermal immunoassay for PSA detection through enzyme-triggered growth of AuNS [94]. Copyright 2019 American Chemical Society.

The in situ growth of plasmonic nanostructures can be effectively combined with enzyme-linked immunoassays based on the reducing ability of enzymatic products, including H_2_O_2_, AA, and others [86,87]. The variation of the LSPR band can result in the differentiable features in photothermal effects [88,89,90]. Wang et al. proposed a photothermal immunoassay for the detection of OTA based on the in situ growth of gold nanobipyramid (AuNBPs) (Figure 5A) [91]. In the absence of OTA, monoclonal antibody (mAb) and secondary antibody-horseradish peroxidase (sAb-HRP) were sequentially immobilized on the OTA-bovine serum albumin (OTA-BSA)-modified microplates. During the classic tyramine signal amplification strategy, HRP catalyzed the oxidation of tyramine by H_2_O_2_, and abundant biotin-tyramine (B-T) molecules were deposited on the plates. Then, a large number of streptavidin-alkaline phosphatase (SA-ALP) conjugates were bound to the plates via the specific interactions between biotin and SA. ALP catalyzed the hydrolysis of L-ascorbic acid 2-phosphotrisodium salt (AAP) into AA. NADP was used to reduce Au(III) to Au(I), which could be reduced into Au(0) by AA and then deposited on the Au seeds to form AuNBPs. After the irradiation of the NIR laser, the solution temperature was significantly increased because of the excellent photothermal conversion efficiency of the in situ formed AuNBPs. The presence of OTA in samples could inhibit in situ growth of AuNBPs and limit the increase in the solution temperature. Gold nanostars (AuNSs) with multiple sharp-branched structures exhibit high photothermal conversion efficiency due to the strong “hotspot” effect, thus facilitating their application in photothermal immunoassays [92]. For example, Wang et al. developed a photothermal immunoassay platform for OTA detection in a combination of tyramine signal amplification, ALP catalysis, and in situ formation of AuNSs [93]. Liu et al. reported a colorimetric and photothermal immunoassay platform for prostate specific antigen (PSA) detection through enzyme-triggered in situ growth of silver shell on AuNSs (Figure 5B) [94]. In this work, the produced AA triggered the deposition of silver onto AuNSs, leading to the growth and formation of silver-coated AuNSs. The silver shell induced the change of dielectric constants and a large blue shift in the LSPR of AuNSs from 770 nm to 540 nm, resulting in the noticeable change of solution color from blue to purple and orange. Meanwhile, the shift of the LSPR absorption peak away from the laser illumination wavelength altered the photothermal conversion efficiency of AuNSs and reduced the change in the environmental temperature. The dual-mode immunoassays showed a low detection limit of 0.95 ng/mL for PSA detection in complex samples.

### 2.2. Carbon-Based Nanostructures

**Figure 6:**
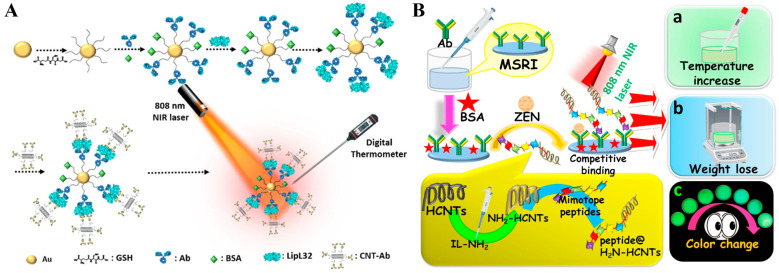
(**A**) Schematic illustration of fabrication of a CNT-based photothermal immunoassay for the detection of LipL32 antigen [96]. Copyright 2024 American Chemical Society. (**B**) Schematic illustration of thermal-responsive hydrogel-based multi-signal readout sensing interface for ZEN detection [97]. Copyright 2020 Elsevier.

Carbon-based materials, including carbon nanotubes (CNTs), graphene oxide (GO), and carbon nano-onion, exhibit strong optical absorption and high photothermal conversion efficiency in the NIR region due to their low cost, unique structure, and high photothermal stability. They have been considered as promising candidates for photothermal therapy, imaging, and photothermal immunoassays (Table 2) [95]. CNTs have attracted considerable interest as photothermal labels due to their high absorption performance in visible-NIR regions and excellent photothermal effect. Sapna et al. reported a photothermal immunoassay for the diagnosis of neglected tropical zoonotic disease using CNTs as labels (Figure 6A) [96]. In this work, GSH-modified AuNPs with higher stability in complex samples were used to load monoclonal antibodies against LipL32 antigen (major outer membrane protein of Leptospira). In the presence of LipL32 antigen and antibody-modified CNTs, the formed sandwich immunocomplexes were separated by centrifugation. Then, the suspension was illustrated with an 808 nm laser, and the obvious increase in temperature was recorded with a thermometer. Stimuli-responsive hydrogels can undergo “smart” multi-signal changes to external stimulus, which are workable for the design of multi-signal immunoassays. For instance, Chen et al. synthesized multi-functional thermal-responsive hydrogels with Cu–Cl mixed ionic liquids and poly (vinyl alcohol) and used them to fabricate a multi-signal readout sensing interface (MSRI) for ZEN detection (Figure 6B) [97]. In this work, helical carbon nanotubes (HCNTs) were modified with amino ionic liquids for the attachment of peptides (peptides@H_2_N-HCNTs). ZEN in samples could compete with the peptides@H_2_N-HCNTs to bind antibodies on MSRI. Under irradiation of an 808 laser, the peptides@H_2_N-HCNTs caused a temperature elevation that could be measured by a portable thermometer. The increased temperature accelerated the color change of Cu–Cl mixed ionic liquids from light blue to green. Moreover, the persistently increased temperature led to weight loss on MSRI, which was recorded by an analytical balance. Based on the mutually corrected signals, the immunoassay for ZEN detection exhibited a linear range from 10^−7^ to 10^−1^ ng/mL with a detection limit of 1.06 × 10^−7^ ng/mL.

**Figure 7:**
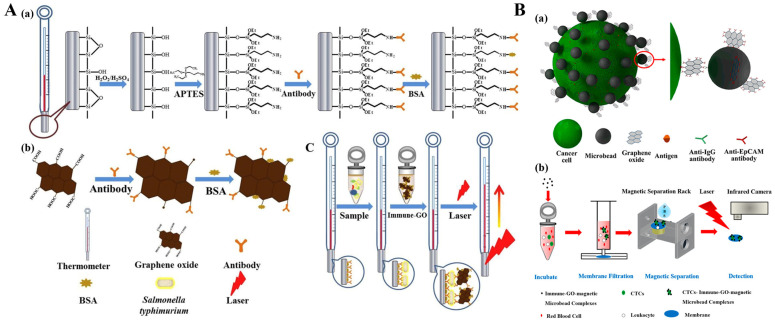
(**A**) Schematic illustration of a portable immune-thermometer assay for the detection of *S. typhimurium* based on the photothermal effect of GO [101]. Copyright 2019 Elsevier. (**B**) Schematic illustration of an immune-GO-magnetic microbead complex for the development of photothermal immunoassay of cancer cell detection [102]. Copyright 2016 American Chemical Society.

GO possesses excellent thermal transport capability and superior photothermal conversion efficiency owing to its full spectrum absorption capacity and large light absorption surface, opening up a new direction in the area of photothermal therapy and immunoassay [98,99,100]. Du et al. developed a portable immune-thermometer method for the detection of *S. typhimurium* based on the photothermal effect of GO (Figure 7A) [101]. To simplify the detection equipment and assay procedure, a common glass thermometer was used as the detector and antibody carrier simultaneously. GO was modified with anti-*S. typhimurium* antibody to produce the immune-GO nanomaterials. After the formation of sandwich complexes on the surface of the thermometer, an increased temperature was generated because of the photothermal effect of GO, which can be directly measured by the thermometer. The detection limit for *S. typhimurium* was found to be 10^3^ CFU/mL, and the entire testing process was consistently completed within 15 min. Taking advantage of magnetic separation by microbeads, Zhang et al. synthesized immune-GO-magnetic microbead complexes for the development of a photothermal immunoassay [102]. As shown in Figure 7B, GO was conjugated with anti-epithelial cell adhesion molecule (EpCAM) antibodies, and magnetic microbeads were modified with anti-IgG antibodies. Through simple mixing and incubation, the immune-GO-magnetic microbead complexes were formed via the anti-EpCAM/anti-IgG interactions, achieving selective recognition of EpCAM antigen on the surface of 4T1 cells. After membrane filtration and magnetic separation to preserve cells and the immune-GO-magnetic microbead complexes, the temperature variation under the irradiation of a laser pen was determined by an infrared camera for the assay of cancer cells.

**Figure 8:**
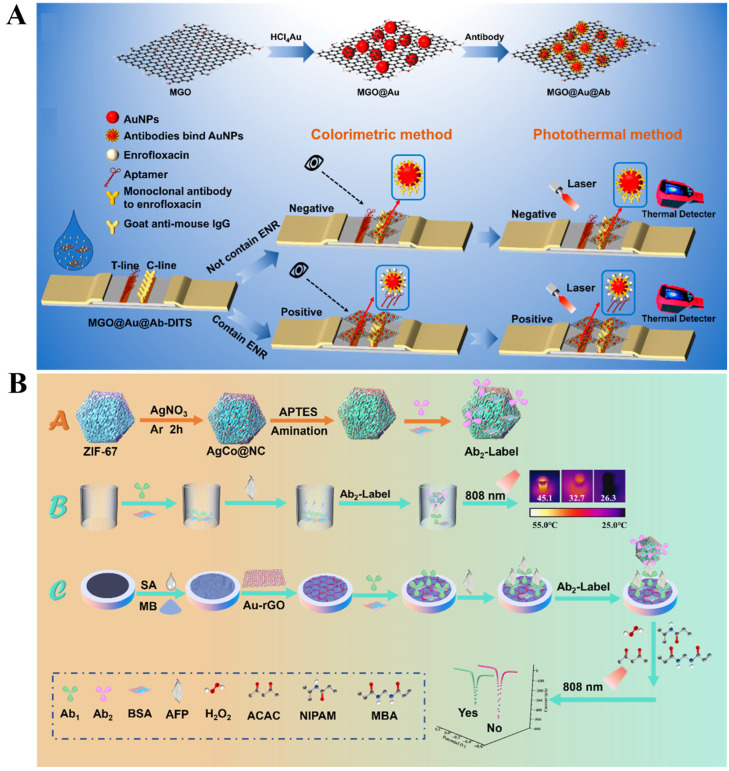
(**A**) Schematic illustration of a MGO@Au-NCs-based photothermal and colorimetric immunochromatographic biosensor for dual-signal detection of ENR in food samples [109]. Copyright 2024 American Chemical Society. (**B**) Schematic illustration of (***A***) preparation of Ab_2_-modified AgCo@NC NP; (***B***) construction of a photothermal immunization platform; (***C***) construction of electrochemical immunization platform [113]. Copyright 2023 American Chemical Society.

Because of its large surface area, abundant functional groups, and aromatic structure, GO can serve as the nanocarrier for the loading of signal molecules or the in situ growth of nanomaterials [103,104]. The combination of other photothermal species with GO can improve the NIR absorption intensity, consequently expanding their photothermal applications, such as AuNRs/GO [105], iron oxide/GO [106], and CuS/GO [107]. In this way, Zhou et al. reported a paper-based immunoassay platform for photothermal detection of cancer cells using GO-AuNPs nanocomposite as photothermal labels [108]. Recently, Liu et al. prepared AuNPs-decorated magnetic GO nanocomposites (MGO@Au-NCs) and developed a photothermal and colorimetric immunochromatographic biosensor for dual-signal detection of enrofloxacin residue (ENR) in food samples (Figure 8A) [109]. In this work, magnetic GO (MGO), a nanohybrid of magnetic NPs and GO, exhibited intrinsic advantages of large specific surface area and paramagnetic/photothermal dual properties. To further improve the photothermal conversion efficiency, MGO was used as the matrix for in situ growth of AuNPs and immobilization of ENR monoclonal antibodies (MGO@Au-NCs@Ab). ENR attached on the aptamer-modified T-line could capture MGO@Au-NCs@Ab. Besides visual observation, MGO@Au-NCs could cause temperature change in the T-line, which was recorded by an infrared camera.

Porous carbon nanomaterials exhibiting sp^2^/sp^3^ carbon hybridization and NIR absorption properties can be synthesized from various templates or precursors through different approaches, such as pyrolysis, carbonization, calcination, and annealing. Among them, pyrolysis of MOFs under an inert atmosphere can lead to the transformation of organic linkers into porous carbon nanomaterials, being endowed with large surface areas, persistent porosities, and controllable functionalities [110]. In addition, metal cations in the MOFs can be concomitantly reduced to form metal nanoparticles on the surface of carbon nanomaterials [111]. The one-pot synthesis of porous carbon/metal nanoparticles nanohybrids offers a facile route toward new photothermal agents with improved photothermal conversion efficiency [112]. For example, Wang et al. constructed a photothermal and electrochemical immunoassay platform for the detection of α-fetoprotein (AFP) based on the photothermal effect of Ag/Co-embedded N-rich mesoporous carbon nanomaterials (AgCo@NC NPs) [113]. As shown in Figure 8B, the AgCo@NC NPs were prepared by successively immersing ZIF-67 into AgNO_3_ solution for chemical reduction, and their photothermal conversion efficiency was calculated to be 46.4%. After calcination under Ar atmosphere, AgCo@NC NPs were modified with a secondary antibody (Ab_2_). The captured AgCo@NC NPs in plates by the immunoreactions could produce obvious temperature change, which was recorded by a hand-held thermograph. Meanwhile, AgCo@NC NPs, as internal heat sources, could accelerate the polymerization of *N*-isopropylacrylamide in the presence of H_2_O_2_ through a Fenton-like reaction. The in situ-formed poorly conductive polymers were deposited on the electrode surface, leading to an increase in impedance and a decrease in signal response. The •OH catalytically produced from H_2_O_2_ could decompose methylene blue, further weakening the current signal.

**Table 2:** Performances of photothermal immunoassays with carbon-based nanostructures as signal labels.

Signal Label	Target	Linear Range	Detection Limit	Ref.
CNTs	LipL32 antigen	0–10^6^ pg/mL	300 fg/mL	[96]
H_2_N-HCNTs	Zearalenone	0.1–10^5^ fg/mL	0.16 fg/mL	[97]
GOs	*S. T*	10^4^–10^8^ CFU/mL	10^4^ CFU/mL	[100]
GOs	*S. T*	10^3^–10^9^ CFU/mL	10^3^ CFU/mL	[101]
GOs	4T1 cell	100–700 cells	100 cells	[102]
GO-Au	MCF-7 cell	Not reported	600 cells	[108]
MGO@Au-NCs	ENR	0–1000 μg/mL	10.89 μg/mL	[109]
AgCo@NC NPs	AFP	0.01–100 ng/mL	3.2 pg/mL	[113]

Abbreviations: CNTs, carbon nanotubes; LipL32 antigen, major outer membrane protein of Leptospira; HCNTs, helical carbon nanotubes; GOs, graphene oxides; *S. T*, *S. typhimurium*; GO-Au, graphene oxide-gold nanocomposite; MGO, magnetic graphene oxide nanoparticles; Au-NCs, Au nanocomposites; ENR, enrofloxacin; AgCo@NC NPs, Ag/Co embedded N-rich mesoporous carbon nanomaterials; AFP, α-fetoprotein.

### 2.3. 2D Nanomaterials

**Figure 9:**
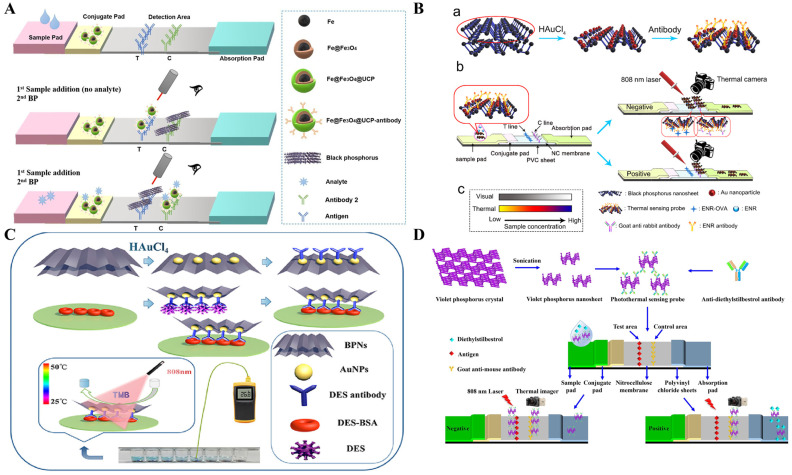
(**A**) Schematic illustration of a black phosphorus-based colorimetric and photothermal immunochromatography for dual-signal detection of NFX [117]. Copyright 2021 Elsevier. (**B**) Schematic illustration of a photothermal LFIA for the detection of veterinary antibiotic enrofloxacin (ENR) based on AuNPs-enhanced BPNSs [122]. Copyright 2019 Elsevier. (**C**) Schematic illustration of a dual-signal immunoassay for diethylstilbestrol detection based on BP-Au nanohybrids and the system of TMB-H_2_O_2_ [123]. Copyright 2021 Elsevier. (**D**) Schematic illustration of an immunochromatographic assay for diethylstilbestrol detection using violet phosphorus nanosheets as photothermal materials [125]. Copyright 2023 Elsevier.

Since the discovery and promising applications of graphene, many 2D nanomaterials with various unique physical and chemical properties have been reported, such as transition metal dichalcogenides (TMDs), hexagonal boron nitride (h-BN), transition metal carbides, nitrides, carbonitrides (MXenes), and layered double hydroxides (Table 3) [114]. Two-dimensional nanomaterials including nanosheets or nanoflakes exhibit much larger light-absorbing surfaces than their 3D counterparts, nanospheres, and nanotubes, and have been widely used as NIR photothermal agents [37]. In particular, black phosphorus nanosheets (BPNSs) exhibit high extinction coefficients and excellent photothermal performance and have aroused considerable interest in photothermal therapy and biosensing [115]. For example, Lu et al. fabricated an immunofiltration strip for 17β-estradiol detection based on the photothermal effect of BPNSs [116]. The BPNSs with a black-brown color in an aqueous solution can serve as color reagents for LFIA. Ren et al. developed a black phosphorus-based colorimetric and photothermal immunochromatography for dual-signal detection of norfloxacin (NFX) (Figure 9A) [117]. In this work, the carboxyl-modified Fe@Fe_3_O_4_@UCP nanocomposites were modified with a monoclonal antibody (mAb) to form a mAb-UCP probe. Antigens were selectively captured by the mAb-UCP probes on the conjugate pad. When the solution flowed through capillary action, the formed immune complexes did not bind to the NFX-BSA antigens on the test line but were captured by the Ab_2_-modified control line. Then, the BPNSs could be captured by the NFX-BSA, and a brown band could be directly observed by naked eyes on the test line. Upon NIR-light illumination, the temperature was recorded through thermal imaging on a mobile phone camera for quantitative analysis. BPNSs can be further assembled with other photothermal materials to boost the photothermal effect and achieve better detection performance than a single nanomaterial [118]. In order to increase the photothermal conversion efficiency, AuNPs were employed to modify BPNSs, and the resulting AuNPs-BPNSs nanocomposites showed enhanced photothermal conversion efficiency for photothermal immunoassays of antigens, such as *S. typhimurium* [119], 17β-estradiol [120], and diethylstilbestrol [121]. Additionally, Li et al. developed a photothermal LFIA platform for the detection of veterinary antibiotic enrofloxacin (ENR) based on AuNPs-enhanced BPNSs [122]. As shown in Figure 9B, AuNPs were in situ formed on the surface of BPNSs, and then antibodies were immobilized on the BP-AuNPs. The color changed from light brown to brown-purple, and the photothermal conversion efficiency of BP was obviously enhanced from 18.79% to 30.77% after coating of AuNPs. By introducing antibody-modified BP-AuNPs into LFIA, ENR was detected with a linear range from 0.03 to 10 μg/L and a detection limit of 0.023 μg/L. Given that the nanocomposites of BPNSs and AuNPs possess enzyme-like properties, Ding et al. developed a dual-signal immunoassay platform for diethylstilbestrol detection based on black phosphorus-gold nanoparticle (BP-Au) nanohybrids and the TMB-H_2_O_2_ system (Figure 9C) [123]. In this work, BP-Au nanohybrids catalyzed the oxidation of TMB into blue color TMBox by H_2_O_2_, providing a colorimetric signal. The photothermal conversion efficiency of BP-Au nanosheets (36.1%) is higher than that of BPNSs alone (23.2%). Meanwhile, both the generated TMBox and the BP-Au nanohybrids possessed excellent photothermal effects to convert the light energy into heat energy, further amplifying the signal of photothermal immunoassays. However, the poor stability of black phosphorus in a water and oxygen system extremely limits the application of such immunoassays in biosensing.

As an allotrope of black phosphorus, violet phosphorus was demonstrated to have the merits of high stability and a wide tunable band gap. It has become a promising candidate for photothermal materials and photocatalysts [124]. Zhang et al. reported a violet phosphorus nanosheets-based photothermal probe for sensitive immunochromatographic sensing of diethylstilbestrol (Figure 9D) [125]. In this work, violet phosphorus nanosheets were prepared through the ultrasonic spalling method, and their photothermal conversion efficiency was found to be 31.1%. The anti-diethylstilbestrol monoclonal antibodies were modified on the nanosheet surface by electrostatic interactions. During a competitive immunochromatographic assay, an increased concentration of diethylstilbestrol led to a decreased amount of photothermal probes on the test area. Under 808 nm laser illustration, violet phosphorus nanosheets induced an increase in temperature that could be recorded by the thermal imager for photothermal detection.

**Figure 10:**
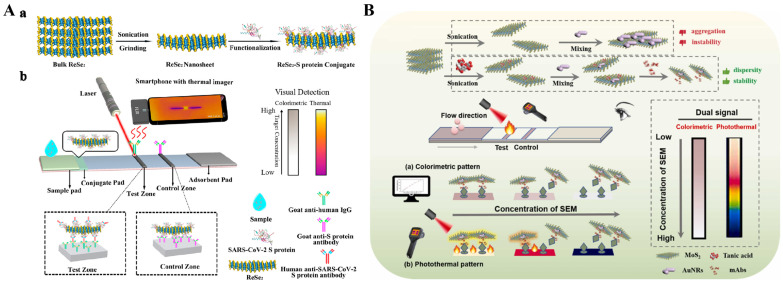
(**A**) Schematic illustration of a smartphone-based photothermal LFIA for the detection of human anti-SARS-CoV-2 S protein IgG antibodies using ReSe_2_ nanosheets [133]. Copyright 2023 American Chemical Society. (**B**) Schematic illustration of an immunochromatography for colorimetric and photothermal detection of nitrofurazone metabolites using the assembly of MoS_2_@AuNRs as the dual-signal probes [138]. Copyright 2023 Elsevier.

Transition metal dichalcogenides (TMDs), such as MoS_2_ and WS_2_, are analogous to graphene but exhibit higher mass extinction coefficients at 800 nm [126]. Thence, TMDs have attracted great attention in photothermal therapy and biosensing [127,128,129,130]. As an example, Du et al. reported a multi-signal dot-filtration immunoassay for the detection of *S. typhimurium* based on the intrinsic color, HRP-like activity, and photothermal effect of MoS_2_ [131]. Transition-metal selenides exhibit similar physicochemical properties with TMDs because of the neighboring position of sulfur and selenium in the periodic table [132]. For this consideration, Hao et al. reported a smartphone-based photothermal LFIA platform for the detection of a human anti-SARS-CoV-2 spike (S) protein IgG antibody using ReSe_2_ nanosheets (Figure 10A) [133]. In this work, ReSe_2_ nanosheets with thicknesses of 10–20 nm were synthesized through wet grinding and ultrasonic probe treatment. They showed a strong absorbance band in the NIR region, a high photothermal conversion efficiency up to 43.2%, and an excellent photothermal stability. The negatively charged ReSe_2_ nanosheets could adsorb the recombinant SARS-CoV-2 S protein through electrostatic interactions to form ReSe_2_-S protein conjugates. S protein IgG antibodies were conjugated with the ReSe_2_-S protein conjugates and then captured by goat antihuman IgG anchored on the detection zone to generate a black line. Excess conjugates were further captured in the control zone to produce the second line. A portable reader was used to collect the colorimetric signals. Besides, under the radiation with a 808 nm laser, the accumulated ReSe_2_ nanosheets in the testing zone generated an increased temperature that could be recorded by a portable thermal imager. The detection limit was calculated to be 0.86 ng/mL. Besides, the combination of TMDs and other photothermal materials can significantly improve the photothermal conversion efficiency of nanocomposites [134,135,136,137]. For example, Yin et al. developed an immunochromatography for colorimetric/photothermal detection of nitrofurazone metabolites by the assembly of AuNRs and MoS_2_ nanosheets (MoS_2_@AuNRs) as the dual-signal probes [138]. As shown in Figure 10B, TA was employed to exfoliate MoS_2_ nanosheets that were further used as the nanocarriers to load AuNRs and provide photothermal and colorimetric dual signals. The photothermal conversion efficiency of TA/MoS_2_@AuNRs was estimated to be around 45.68%. Integrated with a competitive immunochromatography assay, colorimetric signal in the detection area was analyzed by Image J quantitative detection. The temperature change was recorded by the thermal imager view for photothermal quantitative detection. In this method, 2-nitrobenzaldehyde semicarbazone was determined with the detection limits of 0.267 and 0.180 ng/mL, respectively. Furhtermore, Yin’s group synthesized TA-exfoliated WS_2_ nanosheets for competitive immunochromatographic detection of clenbuterol in chicken and skim milk in the same way [139].

**Figure 11:**
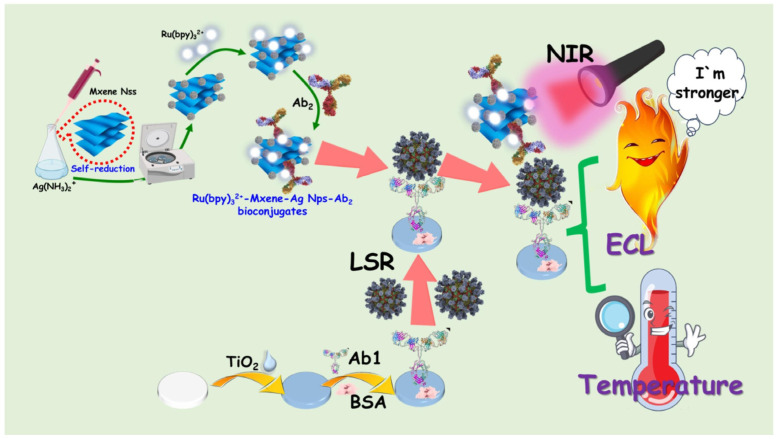
Schematic illustration of an electrochemiluminescence and photothermal immunoassay for the detection of LSR using MXene-based nanocomposite as dual-functional labels [145]. Copyright 2024 Elsevier.

MXenes (e.g., Ti_3_C_2_, Nb_2_C, Ti_2_C, and V_2_C_3_), possessing strong absorbance in the NIR region and excellent photothermal conversion efficiency, have been used as photothermal agents for photothermal therapy and biosensing [140,141]. Moreover, MXenes show high biocompatibility due to the abundant groups on their surface, including fluorine, oxygen, or hydroxyl groups, which are favorable for biomolecule functionalization and application in biosensors [142]. Cai et al. developed a photothermal immunoassay method for PSA detection by using Ti_3_C_2_ MXene quantum dot-encapsulated liposomes with high photothermal conversion efficiency for signal amplification [143]. Liu et al. reported a dual-signal immunosensor for the detection of an autoimmune hepatitis marker based on multifunctional Nb_2_C MXene probes [144]. In this study, the immunoreaction was conducted on the surface of temperature-sensitive polymer poly(N-isopropylacrylamide)-modified electrodes. After the formation of sandwich immunocomplexes, Nb_2_C MXene caused an increase in temperature under 808-nm laser irradiation for photothermal detection. Meanwhile, the attachment of antigens and Nb_2_C MXene conjugates on the electrode surface led to enhanced electrochemical impedance signals. The increased electrode surface temperature resulted in the conformation change of polymers from a free spiral to a spherical shape, further amplifying the impedance signal. The layered nanostructure and versatile surface chemistry of MXenes can promote their integration with nanomaterials to improve photothermal effects. Huang et al. reported an electrochemiluminescence and photothermal immunoassay platform for the detection of Lipolysis-stimulated lipoprotein receptor (LSR) using MXene-based nanocomposites as the dual-functional labels (Figure 11) [145]. In this work, V_2_C MXene served as both the reducing agent and the substrate for in situ growth of AgNPs (V_2_C/Ag) in the presence of Ag(NH_3_)_2_^+^ and then modified with Ru(bpy)_3_^2+^ and antibodies. AgNPs could increase the catalytically active sites and enhance the light absorption of MXene through the LSPR effect. The conversion efficiency for V_2_C/Ag and V_2_C MXene was 27.6% and 26.8%, respectively. Additionally, V_2_C/Ag showed excellent oxygen reduction reaction catalytic activity, greatly amplifying the electrochemiluminescence signal of Ru(bpy)_3_^2+^. Under NIR laser irradiation, V_2_C/Ag effectively converted light energy into heat, leading to an increase in the solution temperature.

**Table 3:** Performances of photothermal immunoassays with 2D nanomaterials as signal labels.

Signal Label	Target	Linear Range	Detection Limit	Ref.
BP	17β-E2	0–20 ng/mL	0.104 ng/mL	[116]
BP	Norfloxacin	0.05–100 ng/mL	45 pg/mL	[117]
BP-Au	DON	1–10^6^ pg/mL	0.154 pg/mL	[118]
BP-Au	17β-estradiol	0.05–50 ng/mL	50 pg/mL	[120]
BP-Au	DES	0.5–50 μg/L	0.1 μg/L	[121]
BP-Au	ENR	0.03–10 μg/L	23 ng/L	[122]
BP-Au	DES	0.0781–20.00 ng/mL	78.1 pg/mL	[123]
VPNS_S_	DES	0.75–50 μg/mL	0.56 μg/mL	[125]
MoS_2_/CuO/Au	CEA	0.1–40 ng/mL	30 pg/mL	[130]
MoS_2_	*S. T*	10^1^–10^7^ CFU/mL	10^2^ CFU/mL	[131]
ReSe_2_	S protein	1–10^4^ ng/mL	0.86 ng/mL	[133]
MoS_2_@Au	*S. T*	10–10^7^ CFU/mL	100 CFU/mL	[135]
MoS_2_@Fe_3_O_4_	*S. T*	10–10^6^ CFU/mL	8 CFU/mL	[136]
TA/MoS_2_@AuNRs	2-NPSEM	Not reported	0.18 ng/mL	[138]
TA-WS_2_	CLE	0–11 ng/mL	0.3 ng/mL	[139]
Ti_3_C_2_ QDs	PSA	1–50 ng/mL	0.4 ng/mL	[143]
Nb_2_C MXene	ASGPR	10^−5^–1 ng/mL	3.3 fg/mL	[144]
V_2_C/Ag	LSR	Not reported	1.53 fg/mL	[145]

Abbreviations: BP, black phosphorus; 17β-E2, 17β-estradiol; BP-Au, black phosphorus-gold nanoparticle; DON, deoxynivalenol; DES, diethylstilbestrol; ENR, enrofloxacin; VPNS_S_, violet phosphorus nanosheets; CEA, carcinoembryonic antigen; *S. T*, *S. typhimurium*; 2-NPSEM, 2-nitrobenzaldehyde semicarbazone; TA-WS_2_, tannic acid assisted stripping WS_2_ nanosheets; CLE, clenbuterol; Ti_3_C_2_ QDs, titanium carbide MXene quantum dots; PSA, prostate specific antigen; ASGPR, autoimmune hepatitis markers; LSR, lipolysis stimulated lipoprotein receptor.

### 2.4. Metal Oxide and Sulfide Nanomaterials

**Figure 12:**
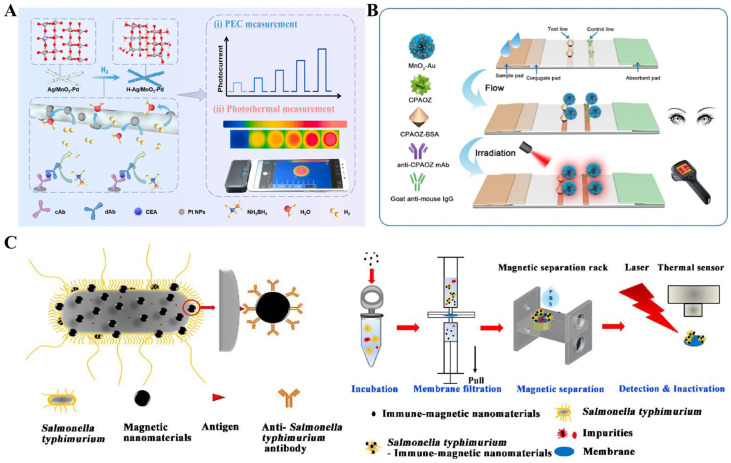
(**A**) Schematic illustration of a photoelectrochemical and photothermal immunoassay for CEA detection based on Ag/MoO_3_–Pd-mediated gasochromic reactions [153]. Copyright 2023 Elsevier. (**B**) Schematic illustration of the colorimetric and photothermal dual-signal immunoassay based on MnO_2_-Au for AOZ detection [154]. Copyright 2022 Elsevier. (**C**) Schematic illustration of a photothermal immunoassay for the detection of *S. typhimurium* based on the photothermal effect of magnetic NPs [157]. Copyright 2022 Elsevier.

In recent years, metal oxide and sulfide nanomaterials have aroused great interest in the biosensing field owing to their advantages of excellent chemical stability, low cost, and unique optical, catalytic, and adsorption properties [146]. Such nanomaterials with excellent photothermal effects have been used as labels or substrates to develop photothermal biosensors (Table 4) [147,148]. For example, molybdenum oxide (MoO_3_), a semiconductor metal oxide nanomaterial with excellent photothermal conversion performance and gasochromic activity, has been extensively used to establish gas sensors with electrical, optical, or photoelectrochemical signals [149,150,151,152]. Lu et al. developed a photoelectrochemical and photothermal immunoassay platform for CEA detection based on Ag/MoO_3_–Pd-mediated gasochromic reactions (Figure 12A) [153]. Herein, Ag-doped MoO_3_ nanorods were synthesized through a hydrothermal method and further modified with Pd NPs and detection antibodies. After the immunoreactions, Pd NPs in the nanocomposites catalyzed the hydrolysis of NH_3_BH_3_ to generate H_2_ that could further be broken into H atoms under the catalysis of Pd NPs. The produced H atoms could react with Ag/MoO_3_ to generate a photoelectrochemical signal. More importantly, the hydrogenated Ag/MoO_3_–Pd exhibited better light absorption properties than Ag/MoO_3_–Pd in the wavelength range of 400–800 nm, and caused an increase in the photothermal conversion efficiency from 3.14% to 20.91%. Under irradiation by an 808 nm laser, the solution temperature was recorded by a near-infrared imaging camera. The dual-mode immunoassay for CEA detection had a detection limit of 26 pg/mL for photoelectrochemical mode and 98 pg/mL for photothermal mode.

Manganese dioxide (MnO_2_) with diverse morphologies and a large surface area can efficiently translate NIR light into thermal energy. Su et al. presented a colorimetric and photothermal immunoassay strategy for the detection of furazolidone using AuNPs-decorated MnO_2_ (MnO_2_-Au) [154]. As shown in Figure 12B, MnO_2_ nanoflowers were used as the nanocarriers to load a large number of AuNPs, greatly boosting the light-to-heat conversion effect. The high absorption of MnO_2_-Au nanocompostes allowed for visual detection by the naked eye. Under NIR irradiation, the photothermal signal was recorded by a thermal infrared imager for quantitative analysis.

Besides magnetic separation and enrichment, magnetic nanomaterials have been popularly utilized in photothermal therapy and biosensors because of their excellent photothermal effect [155,156]. Zhang et al. reported a photothermal immunoassay platform for the detection of *S. typhimurium* based on the photothermal effect of magnetic NPs [157]. As shown in Figure 12C, immune-magnetic NPs were used to capture *S*. *typhimurium* in samples. Then, membrane filtration was carried out to remove the impurities and free immune-magnetic NPs. The nonspecific bacteria were removed by magnetic separation. The magnetic nanomaterials immobilized on the *S. typhimurium*-retained membrane led to the temperature change under irradiation of a laser pen. In addition, Peng et al. developed a sandwich photothermal immunoassay strategy for thyroglobulin detection with magnetic mesoporous CoFe_2_O_4_ labels [158]. In this study, CoFe_2_O_4_ catalyzed the oxidation of TMB to produce TMBox. Under irradiation of an NIR laser, the light–heat conversion was conducted by CoFe_2_O_4_ labels and TMBox, leading to the increase in the solution temperature.

**Figure 13:**
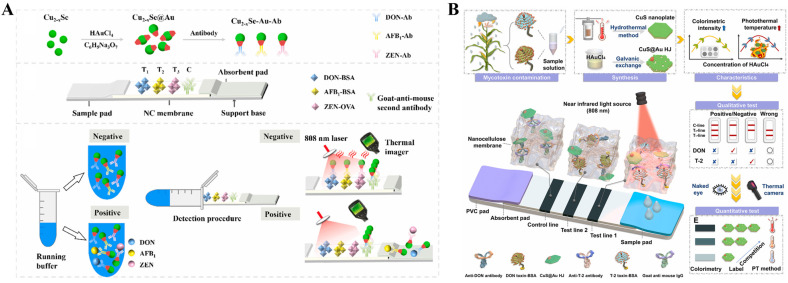
(**A**) Schematic illustration of Cu_2-x_Se-Au nanohybrid-based photothermal LFIA for simultaneous detection of three mycotoxins [166]. Copyright 2023 Elsevier. (**B**) Schematic illustration of dual-plasmonic CuS@Au heterojunctions-based photothermal and colorimetric LFIA for multiplexed detection of T-2 toxin and DON [167]. Copyright 2024 Elsevier.

Copper chalcogenides (Cu_2-x_E, E = S, Se, Te) possess high and stable photothermal conversion efficiency due to the LSPR effect resulting from the copper deficiency. Therefore, it is promising to explore the potential applications of Cu_2-x_S NPs in photothermal imaging, therapy, and biosensing [159,160,161,162,163]. For example, Lv et al. reported a photothermal immunoassay platform for PSA detection based on Cu_x_S nanocrystals and rolling circle amplification [164]. To enhance the photothermal effect and affinity towards proteins, copper chalcogenides have been modified with plasmonic and biocompatible AuNPs [165]. For instance, Huang et al. prepared Cu_2-x_Se-Au nanohybrids as the photothermal labels and developed a LFIA platform for simultaneous detection of three mycotoxins [166]. As shown in Figure 13A, chloroauric acid was reduced into nanogold on the Cu_2-x_Se surface to form Cu_2-x_Se-Au nanocomposites with enhanced photothermal effects. Then, the Cu_2-x_Se-Au nanocomposites were modified with the antibodies of deoxynivalenol (DON), aflatoxin B1 (AFB1), and zearalenone (ZEN), respectively. Targets in samples and artificial antigens on the nitrocellulose membrane could competitively bind with the antibodies conjugated on the nanocomposites. After the competitive immunoassays, the temperature changes induced by the Cu_2-x_Se-Au nanocomposites were measured by a thermal imager. Recently, Shu et al. successfully synthesized dual-plasmonic CuS@Au heterojunctions with synergistically improved photothermal and colorimetric properties for dual-signal multiplexed detection of T-2 toxin and DON [167]. As illustrated in Figure 13B, CuS nanoplates were prepared via a hydrothermal method, and Au nanocrystals were in situ formed on the surface of nanoplates through a galvanic replacement reaction. The coupling of the LSPR effect between CuS nanoplates and Au nanocrystals endowed heterojunctions with enhanced photothermal and colorimetric performances. Compared to CuS nanoplates and Au nanocrystals, the photothermal conversion efficiency of CuS@Au heterojunctions (CuS@Au HJ) (46.8%) increased by 7.22% and 10.16%, respectively. Based on the competitive LFIA platform, T-2 toxin and DON were simultaneously determined with the detection limits of 2.5 and 0.5 ng/mL by the colorimetric mode and 2.5 and 0.1 ng/mL by the photothermal mode, respectively.

**Figure 14:**
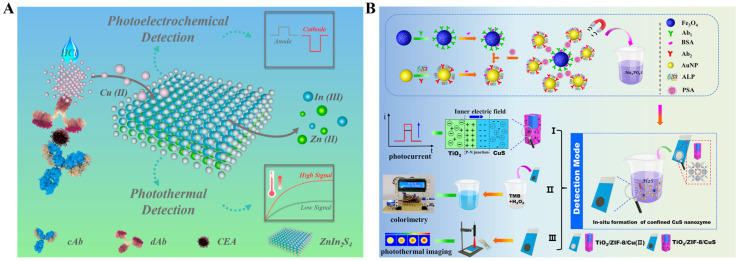
(**A**) Schematic illustration of a photothermal and polarity-switchable photoelectrochemical immunoassay for dual-signal detection of CEA based on cation exchange reaction-mediated in situ generation of CuS [171]. Copyright 2023 American Chemical Society. (**B**) Schematic illustration of a multi-mode immunoassay for PSA detection based on the enzymatic catalysis-induced MOF-confined plasmonic nanozyme [172]. Copyright 2024 American Chemical Society.

The in situ generation of photothermal agents can avoid the influence of biofunctionalization and non-specific adsorption on nanomaterials [168,169,170]. Lu et al. reported a photothermal and polarity-switchable photoelectrochemical immunoassay platform for dual-signal detection of CEA based on cation exchange reaction-mediated in situ generation of CuS [171]. As presented in Figure 14A, detection antibody-labeled CuO NPs were used as the dual-signal probes for sandwich immunoassays. The addition of HCl triggered the release of numerous Cu^2+^ ions and then induced a cation exchange reaction to convert ZnIn_2_S_4_ into CuS. The formed CuS showed a wider light absorption band than ZnIn_2_S_4_, leading to a decreased anode photocurrent from ZnIn_2_S_4_ and an enhanced cathode photocurrent from CuS, thus realizing the polarity-switchable photoelectrochemical detection. Furthermore, the formed CuS exhibited enhanced photothermal conversion efficiency and facilitated the photothermal detection with a digital multimeter. Based on the in situ formation of dual-functional CuS, CEA was accurately detected in the range of 0.1–50 ng/mL (photoelectrochemical mode) and 0.5–100 ng/mL (photothermal mode). Recently, Meng et al. developed a photoelectrochemical, colorimetric, and photothermal triple-signal immunoassay platform for PSA detection based on the enzymatic catalysis-induced MOF-confined plasmonic nanozymes (Figure 14B) [172]. The platform was constructed with the aid of ALP-labeled AuNPs and immunomagnetic beads. After the immunoreaction and magnetic separation, ALP catalyzed the hydrolysis of sodium thiophosphate to release H_2_S that could react with Cu^2+^ to generate CuS in ZIF-8. The formation of p-n heterojunctions (TiO_2_/ZIF-8/CuS) greatly increased the light-harvesting ability and improved the charge separation efficiency, enhancing the photoelectrochemical signal. Besides, CuS as the nanozyme catalyzed the chromogenic reaction of TMB in the presence of H_2_O_2_ for colorimetric detection. More importantly, the formed TiO_2_/ZIF-8/CuS as a photothermal imaging probe showed high photothermal conversion efficiency and led to a significant increase in the temperature under NIR laser irradiation. These multi-mode immunoassays for PSA detection achieved the detection limits of 0.16 pg/mL (photoelectrochemical mode), 0.41 pg/mL (colorimetric mode), and 0.18 pg/mL (photothermal mode).

**Figure 15:**
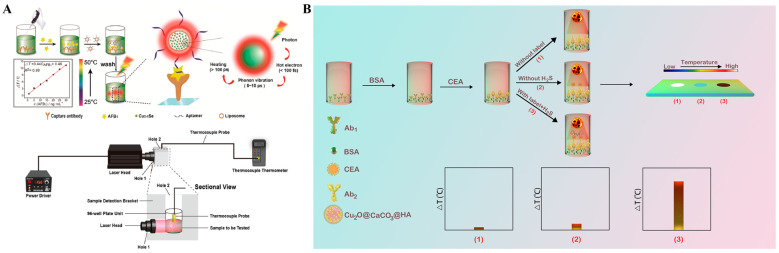
(**A**) Schematic illustration of a photothermal immunoassay of AFB using plasmonic Cu_2-x_Se NPs-loaded liposomes as photothermal soft nanoballs [173]. Copyright 2019 American Chemical Society. (**B**) Schematic illustration of a photothermal immunoassay for CEA detection used based on the in situ transformation of Cu_2_O into Cu_31_S_16_ with photothermal effect [176]. Copyright 2022 American Chemical Society.

Liposomes with a large hydrophilic cavity have been widely applied in the entrapment of signaling molecules and nanomaterials for signal amplification. Li et al. reported the photothermal immunoassay of AFB using plasmonic Cu_2-x_Se NPs-loaded liposomes as photothermal soft nanoballs (Figure 15A) [173]. In this work, liposomes were employed to embed many plasmonic Cu_2-x_Se NPs and then modified with AFB aptamers. After the generation of sandwich structure between the aptamer and the capture antibody on the plate, a large number of Cu_2-x_Se NPs in liposomes could result in the increase of solution temperature under laser irradiation, which was recorded by a thermocouple thermometer. Recently, Liu et al. reported the plasmonic Cu_2-x_Se NPs-liposome-based photothermal immunoassay with a disposable syringe and a filter membrane [174]. The whole testing procedure was easily carried out within 10 min, which was shorter than that of the traditional clinical assays. In addition, Yu et al. reported a photothermal immunoassay strategy for daily detection of cTnI using Cu_2-x_Ag_x_S-loaded liposomes as photothermal labels [175]. Ma et al. constructed a photothermal immunoassay platform for CEA detection based on the in situ transformation of Cu_2_O into Cu_31_S_16_ with photothermal effect (Figure 15B) [176]. In this work, CaCO_3_ shell was growth on the surface of Cu_2_O via the in situ mineralization method to form a Cu_2_O@CaCO_3_ core-shell nanohybrid and sequentially modified with hyaluronic acid and secondary antibodies. The target CEA in samples could lead to the formation of sandwich immunocomplexes. Cu_2_O was converted into Cu_31_S_16_ by reacting with H_2_S, greatly enhancing the photothermal conversion efficiency in the NIR II region (1000–1350 nm). Besides, the formed Cu_31_S_16_ NPs with good electron transport properties could amplify the electrical signal of redox pairs for electrochemical detection.

**Table 4:** Performances of photothermal immunoassays based on metal oxide and sulfide nanomaterials.

Signal Label	Target	Linear Range	Detection Limit	Ref.
NiS_2_	NFZ	0–0.1 μg/kg	0.01 μg/kg	[148]
Ag/MoO_3_-Pd	CEA	0.1–100 ng/mL	98 pg/mL	[153]
MnO_2_-Au	FZD	0.1–1 ng/mL	0.43 ng/mL	[154]
Magnetic NMs	*S. T*	300–1000 CFU/mL	300 CFU/mL	[157]
CoFe_2_O_4_	TG	0.01–10 ng/mL	8.3 pg/mL	[158]
Cu_x_S	PSA	1–200 ng/mL	0.2 ng/mL	[164]
Cu_2-x_Se-Au	DON	0.1–200 ng/mL	73 pg/mL	[166]
CuS@Au	DON	20–60 ng/mL	2.5 ng/mL	[167]
INH-Ag	CEA	0.1–40 ng/mL	80 pg/mL	[170]
ZnIn_2_S_4_	CEA	0.5–100 ng/mL	0.21 ng/mL	[171]
TiO_2_/ZIF-8/Cu(II)	PSA	10^−12^–10^−7^ g/mL	0.16 pg/mL	[172]
Cu_2−x_Se NCs	AFB1	1–30 ng/mL	0.19 ng/mL	[173]
Cu_2-x_Se@liposome	CEA	0–50 ng/mL	97 pg/mL	[174]
Cu_2−x_Ag_x_S@liposome	cTnI	0.02–10 ng/mL	11.2 pg/mL	[175]

Abbreviations: NFZ, nitrofurazone; Ag/MoO_3_–Pd, Ag-doped/Pd nanoparticles loaded MoO_3_ nanorods; CEA, carcinoembryonic antigen; FZD, furazolidone; NMs, nanomaterials; *S. T*, *S. typhimurium*; TG, thyroglobulin; PSA, prostate specific antigen; DON, deoxynivalenol; NCs, nanocrystals; AFB1, aflatoxin B1; INH–Ag, azide-modified Ag nanoparticles; cTnI, cardiac troponin I.

### 2.5. Prussian Blue-Based Materials

**Figure 16:**
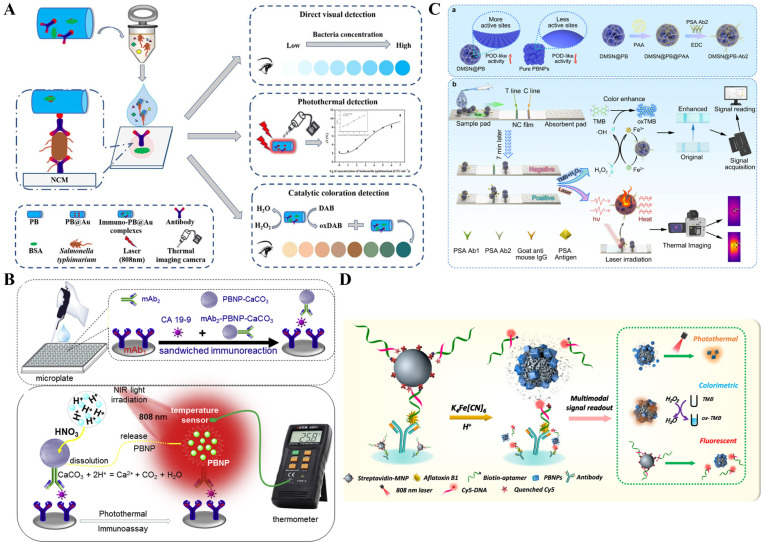
(**A**) Schematic illustration of a *S. typhimurium* strip based on the photothermal effect and catalytic color overlap of PB@Au nanocomposite [119]. Copyright 2022 Elsevier. (**B**) Schematic illustration of a photothermal immunoassay for pancreatic cancer biomarker CA 19-9 using PBNPs-encapsulated CaCO_3_ microspheres as signal labels [184]. Copyright 2020 Elsevier. (**C**) Schematic illustration of a colorimetric and photothermal LFIA for PSA detection based on forcibly dispersed PBNPs [185]. Copyright 2024 American Chemical Society. (**D**) Schematic illustration of an in situ formed PBNPs-mediated multi-signal nanozyme-linked immunosorbent assay for AFB1 detection [191]. Copyright 2021 American Chemical Society.

As a family of common inorganic nanomaterials, PBNPs show remarkable advantages of simple synthesis, low cost, and excellent biocompatibility. They possess peroxidase-like (POD-like) activity and have a wide range of applications as nanozymes in bioassays [177,178]. In recent years, PBNPs have been successfully utilized as photothermal agents in immunoassays due to their high molar extinction coefficient (1.09 × 10^9^ mol L^−1^ cm^−1^) and excellent photothermal conversion efficiency (18.7%) in the NIR region (Table 5) [179,180,181]. It is a simple and straightforward strategy to use PBNPs as the photothermal labels in immunoassays [182]. For example, Hong et al. developed a photothermal immunoassay platform for the determination of human chorionic gonadotropin based on PBNPs-mediated photothermal conversion under 980-nm laser irradiation [183]. Lu et al. constructed a *S. typhimurium* strip based on the photothermal effect and catalytic color overlap of PB@Au nanocomposites [119]. As presented in Figure 16A, AuNPs were in situ deposited on the surface of PBNPs and further modified with the anti-*S. typhimurium* antibodies. After the label and capture of *S. typhimurium*, PB@Au nanocomposites in the immune complexes could cause an obvious temperature increase. Besides its intrinsic blue color, PBNPs could catalyze the oxidation of 3,3′-diaminobenzidine tetrahydrochloride (DAB) into its oxidation state (oxDAB), and the absorption band of brown oxDAB overlaps with that of the blue PBNPs to form multicolor, from light red to brown-red and then to blue-gray. To amplify the signal and reduce the detection limit, Han et al. reported the photothermal immunoassay of pancreatic cancer biomarker CA 19-9 by using calcium carbonate (CaCO_3_) to encapsulate PBNPs as the signal labels (Figure 16B) [184]. PBNPs were embedded in CaCO_3_ via a reverse micelle method and then modified with anti-CA 19-9 antibodies to form mAb_2_-PBNP-CaCO_3_ bioconjugates. In the presence of CA 19-9 targets, the immunocomplexes were formed on the microplates. After the addition of the acidic solution, the immobilized CaCO_3_ microspheres were destroyed, releasing a large number of PBNPs. Under irradiation of an 808 nm laser, the released PBNPs could induce a temperature shift, which was related to the concentration of CA 19-9, and the signal could be recorded by a portable digital thermometer. Recently, Gong et al. reported a colorimetric and photothermal LFIA method for PSA detection based on the forcibly dispersed PBNPs (Figure 16C) [185]. The PBNPs nanozymes were in situ grown within the pores of dendritic mesoporous silica (DMSN, DMSN@PB), leading to an increase in the exposed active sites. The POD-like activity was higher than that of cubic PBNPs due to the enhanced atom utilization efficiency. The antibody-modified DMSN@PB was used in the sandwich immunoassay. PBNPs could catalyze the oxidation of TMB into blue TMBox and enhance the colorimetric response of bright blue colored PBNPs. Meanwhile, PBNPs provided a photothermal signal for PSA detection. The dual-signal immunoassays achieved the detection of PSA with a detection limit of 0.520 ng/mL for colorimetric mode assay and 0.202 ng/mL for photothermal assay.

The in situ growth of PBNPs is a promising strategy to combine immunoassay with photothermal detection [39,186,187]. In 2016, Fu et al. first reported a photothermal immunoassay for PSA detection based on the in-situ transformation of Fe_3_O_4_ NPs into PBNPs [188]. Later, several in situ formations of PBNPs-based photothermal immunoassays were developed for the detection of PSA [189,190]. For example, Lu et al. reported an in situ formed PBNPs-mediated multi-signal nanozyme-linked immunosorbent assay for AFB1 detection [191]. As illustrated in Figure 16D, AFB1 aptamer-modified magnetic nanoparticles (MNPs) were used to capture AFB1 in samples and were then captured by the antibody-modified 96-well plates. In the presence of HCl and K_4_Fe(CN)_6_, Fe^3+^ ions released from MNPs were reacted with K_4_Fe(CN)_6_ to in situ produce PBNPs on the MNPs surface, which could convert light energy into heat energy for photothermal detection. The formed PBNPs with intrinsic POD-like activity catalyzed the oxidization of colorless TMB to blue color TMBox for colorimetric detection. Moreover, the fluorescence of Cy5-labeled DNA quenched by MNPs was recovered after the etching of MNPs and the formation of PBNPs, realizing the fluorescence detection of AFB1.

**Table 5:** Performances of photothermal immunoassays with PB-based materials as signal labels.

Signal Label	Target	Linear Range	Detection Limit	Ref.
PBNP	NSE	0.1–100 ng/mL	53 pg/mL	[182]
PBNP	HCG	0.01–100 ng/mL	5.8 pg/mL	[183]
PBNP-CaCO_3_	CA 19-9	1–100 U/mL	0.83 U/mL	[184]
DMSN@PB	PSA	1–40 ng/mL	0.202 ng/mL	[185]
PBNP	PSA	2–64 ng/mL	1 ng/mL	[188]
PBNP	AFB1	10^−4^–10^4^ ng/mL	3.42 pg/mL	[191]

Abbreviations: PBNP, Prussian blue nanoparticles; NSE, neuron-specificenolase; HCG, human chorionic gonadotropin; PBNP-CaCO_3_, Prussian blue nanoparticles-encapsulated CaCO_3_; CA 19-9, carbohydrate antigen 19-9; DMSN@PB, Prussian blue within the pores of dendritic mesoporous silica; PSA, prostate specific antigen; AFB1, aflatoxin B1.

### 2.6. Small Organic Molecules

**Figure 17:**
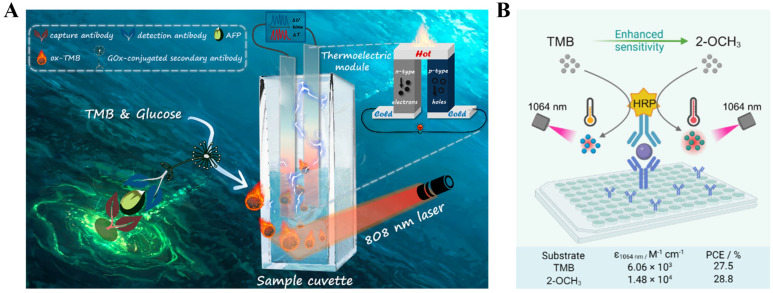
(**A**) Schematic illustration of the photothermal-thermoelectric coupled immunoassay for AFP detection with a self-powered temperature sensor [199]. Copyright 2020 American Chemical Society. (**B**) Schematic illustration of a 1064 nm-excited photothermal immunoassay for PSA detection based on NIR-II-absorbing TMB derivative as the HRP substrate [201]. Copyright 2024 American Chemical Society.

Organic NIR dyes have been widely used in photothermal therapy, but their application in photothermal immunoassays was limited because of the poor photothermal conversion efficiency [192,193]. During the past decade, several organic chromogenic molecules used for colorimetric assays, such as indocyanine green, *o*-phenylenediamine, and ABTS, have been proposed to develop photothermal bioassays because their oxidative products exhibit relatively high photothermal conversion efficiency (Table 6) [194,195]. As the product of the enzyme/nanozyme-catalyzed colorimetric reaction, TMBox shows an increased absorption intensity in the NIR region [196]. Thus, it is feasible to combine the TMB-generated enzymatic colorimetric system with NIR laser-driven photothermal immunoassay [197,198]. For example, Huang et al. constructed a self-powered temperature sensor with Seebeck effect transduction for photothermal-thermoelectric combined immunoassays (Figure 17A) [199]. With the change in temperature, the closed loop formed by two conductive materials can produce distinct potentials, which was called the Seebeck effect. In this study, glucose oxidase (GOx) conjugated with a detection antibody could catalyze the oxidation of glucose and produce H_2_O_2_ to oxidate TMB. The generated blue TMBox with excellent photothermal effect could cause a great increase in temperature that was then converted into an electrical signal via the flexible thermoelectric module in a 3D-printed integrated device. The photothermal-thermoelectric immunoassay for AFP detection exhibited a dynamic linear range from 0.5 to 60 ng/mL with a detection of 0.39 ng/mL. The molar absorption coefficients of TMBox were 1.2 × 10^4^ M^−1^ cm^−1^ at 808 nm and 6.06 × 10^3^ M^−1^ cm^−1^ at 1064 nm, respectively. However, the relatively low absorption coefficient may limit the sensitivity of photothermal bioassays. It is noticed that the absorption of water in the NIR-I window (700~900 nm) is strong, but it is relatively low in the NIR-II window (1000~1700 nm) [200]. Thus, it is important to synthesize photothermal materials with a maximum photothermal conversion efficiency in the NIR-II region. Recently, Liu et al. prepared a NIR-II-absorbing TMB derivative for photothermal immunoassays with a 1064 nm laser (Figure 17B) [201]. The derivative 3,3′-dimethoxy-5,5′-dimethylbenzidine (2-OCH_3_) was designed by introducing electron-donating methoxy groups in the TMB framework to narrow the band gap between the highest occupied molecular orbital (HOMO) and the lowest unoccupied molecular orbital (LUMO). The red-shifted absorption of 2-OCH_3_ resulted in an enhanced molar absorption coefficient of 1.48 × 10^4^ M^−1^ cm^−1^ at 1064 nm within the NIR-II region and an increased photothermal efficiency (28.8%). In a conventional HRP-labeled ELISA using 2-OCH_3_ as the substrate, the 1064 nm-excited photothermal immunoassay for PSA detection achieved a detection limit of 0.1 ng/mL and showed an enhanced sensitivity over TMB-based immunoassay. However, natural enzymes in conventional ELISAs exhibit several undesired shortcomings, including short shelf life, high cost, and weak stability against harsh environments.

**Figure 18:**
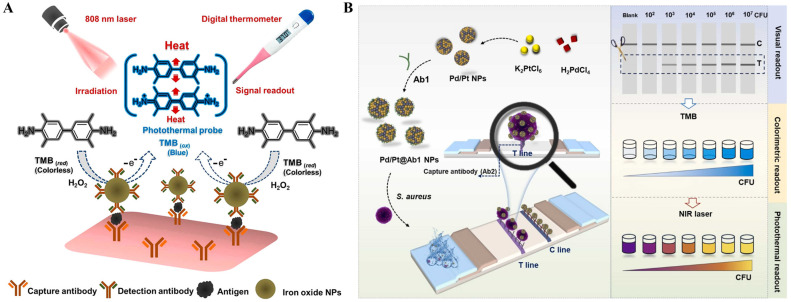
(**A**) Schematic illustration of the photothermal and colorimetric immunoassay based on the photothermal effect of the iron oxide NPs-mediated TMB-H_2_O_2_ colorimetric system [206]. Copyright 2018 American Chemical Society. (**B**) Schematic illustration of a multi-signal LFIA for the detection of *S. aureus* using bimetallic Pd/Pt NPs [220]. Copyright 2024 Elsevier.

Since the intrinsic peroxidase-like activity of ferromagnetic (Fe_3_O_4_) nanoparticles was reported in 2007 [202], nanozymes with different enzyme-like catalytic properties have been synthesized and extensively applied in immunoassays as alternatives to natural enzymes [203,204,205]. Compared with natural enzymes, nanozymes possess unique merits of high stability against harsh environments, low cost, easy synthesis, and tunable catalytic activity. Fu et al. firstly reported a colorimetric and photothermal immunoassay strategy based on the iron oxide NPs-mediated photothermal effect with the TMB-H_2_O_2_ system [206]. As presented in Figure 18A, the iron oxide NPs catalyzed the oxidation of TMB into TMBox by H_2_O_2_, leading to the solution color change from colorless to blue. Due to the increased absorbance from 750 nm, TMBox could cause an obvious temperature rise under the irradiation of an 808 nm laser. The method can determine 1 ng/mL PSA in normal human serums. After that, photothermal assays of different targets based on the nanozymes or artificial enzymes-catalyzed generation of TMBox have been reported, including metal ions, glucose, dopamine, ENR, pyrophosphatase, cancer cells, and *S. Typhimurium* [207,208,209,210,211,212,213,214,215,216,217,218,219]. Recently, Liu et al. reported a multi-signal LFIA for the detection of *Staphylococcus aureus (S. aureus)* using bimetallic palladium/platinum nanoparticles (Pd/Pt NPs) [220]. As shown in Figure 18, Pd/Pt NPs were synthesized via a one-pot method and then modified with detection antibodies. The monoclonal capture antibody of *S. aureus* anchored on the NC membrane allowed for the capture of *S. aureus* and detection of antibody-modified Pd/Pt NPs, producing a gray color on the T-line. Pd/Pt NPs on the T-line served as nanozymes to catalyze the oxidation of TMB into blue color TMBox for colorimetric analysis. Furthermore, the generated TMBox resulted in a temperature increase for photothermal detection of *S. aureus*. To avoid the use of unstable H_2_O_2_, Lian et al. reported a colorimetric and photothermal immunosensor for chloramphenicol detection based on the β-NiOOH nanozyme and TMB system [221]. Noble-metal-free transition-metal hydroxide β-NiOOH with high oxidase-like activity could catalyze the transformation of colorless TMB into blue TMBox in the absence of H_2_O_2_, producing colorimetric and photothermal signals for competitive immunoassays.

**Figure 19:**
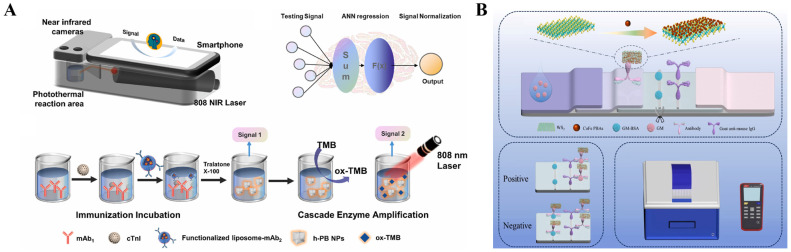
(**A**) Schematic illustration of the constructed portable photothermal-colorimetric dual-modality biosensor for cTnI detection [227]. Copyright 2022 Elsevier. (**B**) Schematic illustration of CoFe PBAs/WS_2_ nanozyme-based chemiluminescence and photothermal dual-signal LFIA for sensitive detection of gentamicin [228]. Copyright 2024 Elsevier.

In recent years, nanozymes with both catalytic ability and photothermal effect have been explored for photothermal catalytic applications [222], such as copper-doped melanin nanozymes [223], hemin–rGO@Au NFs [224], Fe-N-C single-atom nanozymes [225], and Au@CeO_2_ hybrid nanozymes [226]. In addition, PB and its analogs (PBA) with good peroxidase-like activity and excellent photothermal conversion efficiency exhibit a great potential for colorimetric and photothermal biosensors. Meanwhile, liposome encapsulation has become a popular signal-amplified strategy for various bioassays. For this view, Yu et al. developed a multimodal colorimetric-photothermal immunoassay platform for the detection of cTnI protein by using hollow PB (h-PB)-encapsulated liposomes (Figure 19A) [227]. Compared with PB, h-PB exhibited higher peroxidase activity due to the larger specific surface area and more abundant reactive sites. Liposome was used to encapsulate h-PB and anchor the detection antibodies. After the immunoreaction, tralatone-X100 was added to trigger the release of numerous photothermal h-PB signal beacons. In the presence of H_2_O_2_, the released h-PB catalyzed the oxidation of TMB into blue product TMBox. Both TMBox and h-PB possessed excellent photothermal conversion efficiency. The photothermal conversion experiments were conducted under irradiation of an 808 nm NIR laser to provide the temperature signal for cTnI detection. The artificial neural network was applied in bimodal signal processing and regression. This immunosensor realized the detection of cTnI with a dynamic range of 0.02–20 ng/mL and a detection limit of 10.8 pg/mL. In addition, anchoring of PBNPs on other materials has huge potential applications for POCT immunoassays. Recently, Wu et al. reported a chemiluminescence and photothermal dual-signal LFIA strategy for sensitive detection of gentamicin by using CoFe PBAs/WS_2_ nanozymes (Figure 19B) [228]. In this study, the peroxidase-like activity of CoFe PBAs/WS_2_ nanocomposites was greatly enhanced due to the doping effect of Co and the synergistic effect between WS_2_ and CoFe PBAs. The CoFe PBAs/WS_2_ nanozymes could effectively catalyze the oxidation of luminol in the presence of H_2_O_2_, producing an enhanced chemiluminescence signal. Meanwhile, the nanozymes could catalyze the oxidation of TMB by H_2_O_2_. Both the generated TMBox and CoFe PBAs/WS_2_ nanozymes showed excellent photothermal effect and caused the temperature change under laser irradiation. This LFIA platform in the chemiluminescence and photothermal modes showed quantification ranges of 0.001–100 and 0.05–100 ng/mL with detection limits of 0.33 and 16.67 pg/mL, respectively.

**Figure 20:**
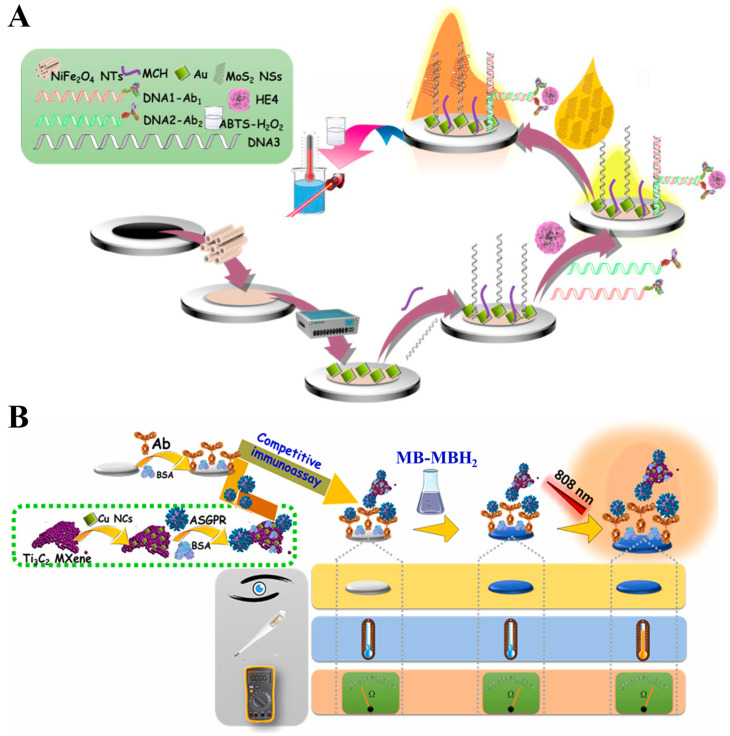
(**A**) Schematic illustration of a split-type proximity hybridization-based photothermal and electrochemiluminescence immunoassay for HE4 detection based on MoS_2_ NSs and ABTS [229]. Copyright 2020 Elsevier. (**B**). Schematic illustration of a portable multi-signal immunoassay for the detection of human anti-ASGPR based on plasmonic MXene-induced signal amplification [230]. Copyright 2022 Elsevier.

Like TMB, 2,2′-azinobis(3-ethylbenzothiazoline)-6-sulfonic acid (ABTS) is a widely used chromogenic substrate in colorimetric bioassays. Zhang et al. reported a split-type proximity hybridization-based photothermal and electrochemiluminescence immunoassay method for the detection of human epididymis-specific protein 4 (HE4) based on MoS_2_ NSs and ABTS [229]. As presented in Figure 20A, the electrode was modified with NiFe_2_O_4_ nanotubes and then electrochemically deposited with AuNPs. The presence of HE4 triggered the immunological recognition between HE4 and DNA-linked antibody, facilitating the proximity hybridization. Then, MoS_2_ NSs were immobilized onto the electrode surface by the Van der Waals force between MoS_2_ NSs and ss-DNA. MoS_2_ NSs with POD-like activity served as the co-reaction accelerators in the luminol-O_2_ system and produced an amplified electrochemiluminescence signal. Meanwhile, MoS_2_ NSs catalyzed the oxidation of ABTS to green-colored ABTS^•+^ in the presence of H_2_O_2_. Both MoS_2_ NSs and ABTS^•+^ had a photothermal effect and caused a temperature increase under laser irradiation for photothermal detection. Chen et al. developed a portable multi-signal immunoassay platform for the detection of human anti-asialoglycoprotein receptor (anti-ASGPR) based on plasmonic MXene-induced signal amplification (Figure 20B) [230]. The photothermal conversion efficiency of Ti_3_C_2_ MXene@CuNCs (25.17%) was obviously higher than that of pure Ti_3_C_2_ MXene nanosheets (15.03%). The plasmonic Ti_3_C_2_ MXene@CuNCs were used and modified with anti-ASGPR antigen and acted as the photothermal probes. After the competitive immunoassays, Ag-Ti_3_C_2_ MXene@CuNCs immobilized on the soft electronic device could catalyze the oxidation of the reduced methylene blue (MBH_2_) to methylene blue (MB), which was accompanied by the solution color change from moderated blue to deep blue. Moreover, the formed Ti_3_C_2_ MXene@CuNCs/MB complexes exhibited an enhanced photothermal effect. Under NIR laser irradiation, the temperature elevation was recorded by a portable thermometer.

**Table 6:** Performances of organic molecules-assisted photothermal immunoassays.

Signal Label	Target	Linear Range	Detection Limit	Ref.
Ag_2_CO_3_@Ag	CEA	0.1–5 ng/mL	0.08 ng/mL	[193]
Ti_3_C_2_T_x_/AuNPs	ZEN	0.5–500 ng/L	0.48 pg/mL	[197]
Pt-CN	AFB_1_	1–10^4^ pg/mL	0.76 pg/mL	[205]
Fe_3_O_4_ NPs	PSA	1.0–64 ng/mL	1 ng/mL	[206]
BP-Pt	ENR	0.01–100 ng/mL	8.3 pg/mL	[207]
PtNPs	YKL-40	0.03–100 ng/mL	14 pg/mL	[208]
PtNiCo@TA	*S. T*	5 × 10^2^–5 × 10^4^ CFU/mL	5 × 10^2^ CFU/mL	[219]
Pd/Pt	*S. aureus*	10^2^–10^7^ CFU/mL	4 CFU/mL	[220]
2-OCH_3_	PSA	0–20 ng/mL	0.1 ng/mL	[201]
NiOOH	CAP	0–5 ng/mL	1.6 pg/mL	[221]
h-PB	cTnI	0.02–20 ng/mL	10.8 pg/mL	[227]
CoFe PBAs/WS_2_	GM	0.05–100 ng/mL	16.67 pg/mL	[228]
MoS_2_	HE4	10^−4^–10 ng/mL	35 fg/mL	[229]
Ti_3_C_2_ MXene@CuNCs	anti-ASGPR	10^−8^–10^−3^ U/mL	1.19 × 10^−8^ CFU/mL	[230]

Abbreviations: CEA, carcinoembryonic antigen; ZEN, zearalenone; Pt-CN, Pt supported on nitrogen-doped carbon amorphous; AFB1, aflatoxin B1; PSA, prostate specific antigen; BP-Pt, black phosphorus-platinum two-dimensional nanomaterials; ENR, enrofloxacin; PtNPs, Platinum nanoparticles; YKL-40, chitinase-3-like protein 1; PtNiCo@TA, tannic acid-modified PtNiCo trimetallic nanoflower; *S. T*, *S. typhimurium*; 2-OCH_3_, 3,3′-dimethoxy-5, 5′-dimethylbenzidine; CAP, chloramphenicol; h-PB, hollow Prussian blue nanoparticles; cTnI, cardiac troponin I; PBAs, Prussian blue analogs; GM, gentamicin; HE4, human epididymis-specific protein 4; CuNCs, copper nano-clusters; anti-ASGPR, anti-asialoglycoprotein receptor.

### 2.7. Polymers

**Figure 21:**
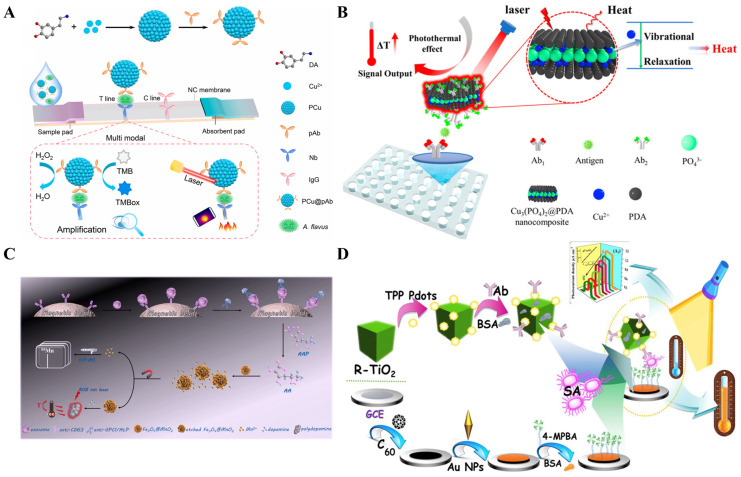
(**A**) Schematic illustration of the preparation of the PCu@pAb probe and sandwich mechanism for the peroxidase-like colorimetric and photothermal multimodal analysis of *A. flavus* [232]. Copyright 2019 American Chemical Society. (**B**) Schematic illustration of the photothermal immunosensor using the Cu_3_(PO_4_)_2_/PDA nanosheets [234]. Copyright 2019 American Chemical Society. (**C**) Schematic illustration of an ICP-MS and photothermal immunoassay for the detection of exosomes based on the bioetching of MnO_2_ shell and in situ generation of PDA [236]. Copyright 2021 American Chemical Society. (**D**) Schematic illustration of a TPP-Pdots-based photoelectrochemical and photothermal immunoassay for sensitive detection of sialic acid [237]. Copyright 2019 Elsevier.

As an important component of melanin, polydopamine (PDA) formed by the polymerization of dopamine (DA) is widely distributed in the human body. The polymer of PDA has a broad-band absorption ability in the UV-Vis to NIR regions and can efficiently convert NIR light into heat. It has been popularly used as the photothermal agent for cancer therapy. Besides, abundant active groups such as hydroxyl and amine, and plentiful aromatic rings on the PDA surface make it possible to carry antibodies and other species by the covalent or π–π stacking interactions. Thus, PDA is a promising photothermal agent for the development of photothermal immunoassays (Table 7) [231]. The catechol units in PDA with high affinity to different metal ions can accelerate the formation of metal nanomaterials on its surface through the reduction of metal ions without the use of extra reducing agents. Thus, PDA has been used as an effective template for the fabrication of different metal nanostructures. For example, Liang et al. developed a colorimetric and photothermal LFIA platform for *Aspergillus flavus* detection using a compact Cu-anchored PDA (PCu) as the signal label [232]. As shown in Figure 21A, the PCu assemblies were readily prepared via a one-step DA-mediated polymerization and in situ doping and reduction procedure. After the modification with *A. flavus*-specific recognition rabbit polyclonal antibody (pAb), PCu@pAb was used to capture *A. flavus*. When PCu@pAb was immobilized on the nanobody (Nb)-modified T-line, its characteristic gray color produced a colorimetric signal. Meanwhile, the PCu assemblies with POD-mimicking properties could catalyze the chromogenic reaction between TMB and H_2_O_2_, producing an obvious blue color and amplified colorimetric signal. In addition, PDA with photothermal effect could lead to a high temperature signal.

Dopamine molecules can self-polymerize into thin, surface-adherent films on the surface of different materials, such as noble metals, oxides, and semiconductors [233]. Tan et al. prepared PDA-coated Cu_3_(PO_4_)_2_ nanosheets (Cu_3_(PO_4_)_2_@PDA) for photothermal immunoassays (Figure 21B) [234]. In this study, Cu_3_(PO_4_)_2_ had a large light-absorbing surface and high biocompatibility and biodegradability. The authors found that the photothermal conversion efficiency of Cu_3_(PO_4_)_2_ and Cu_3_(PO_4_)_2_@PDA was 22% and 45.2%, respectively. Thus, the coating of PDA on Cu_3_(PO_4_)_2_ nanosheets led to an increase in the photothermal conversion efficiency by more than two-fold. The value is higher than that of other PDA-containing photothermal agents, including PDA-coated Fe_3_O_4_ (13.1%), PDA/Au hollow superparticles (38%), and dopamine-melanin colloidal nanospheres (40%). It is also greater than that of Cu-based photothermal agents, such as flowerlike copper phosphate (41%), copper selenide nanocrystals (22%), and copper sulfide nanocrystals (16.3%) [234]. More importantly, Cu_3_(PO_4_)_2_ could enhance the activity and stability of antibodies. After coating with PDA shell, the photothermal conversion efficiency of Cu_3_(PO_4_)_2_ was significantly improved, and the positively charged PDA shell could electrostatically adsorb the secondary antibody (Ab_2_) of CRP. After the formation of immunocomplexes and irradiation by an 808 nm NIR laser, the temperature change of the solution was measured by a pen-style thermometer. Similarly, Yang et al. reported a colorimetric and photothermal LFIA method for dual-signal detection of *S. typhimurium* using PDA-coated Cu_2_MoS_4_ nanosheets as the photothermal signal tags [235]. The introduction of PDA not only enhanced the photothermal conversion efficiency of Cu_2_MoS_4_ nanosheets and the water solubility but also provided abundant functional groups for the attachment of antibodies. The synthesized PDA-coated Cu_2_MoS_4_ nanosheets showed black color for visual detection and exhibited improved photothermal conversion efficiency for photothermal detection.

Metal ions or nanomaterials with oxidative activity can catalyze the polymerization of DA into PDA through the photothermal effect [238]. Zhang et al. reported the inductively coupled plasma mass spectrometry (ICP-MS) and photothermal immunoassays of exosomes based on the bioetching of MnO_2_ shell and the in situ generation of PDA [236]. As shown in Figure 21C, the ALP-linked sandwich immunoassay was conducted with immune-magnetic beads. ALP catalyzed the hydrolysis of AAP to generate AA that could etch Fe_3_O_4_@MnO_2_ nanoflowers to release Mn^2+^. The generated Mn^2+^ in the supernatant was determined by ICP-MS. After magnetic separation, the etched Fe_3_O_4_@MnO_2_ nanoflowers could further oxidize DA into PDA with an excellent NIR-driven photothermal effect. The obtained ICP-MS and photothermal signals reflected the number of exosomes in samples.

Polypyrrole nanoparticles, polymerized from pyrrole, have been used as NIR photothermal probes because of their high photothermal conversion efficiency and excellent photothermal stability [239]. Zhang et al. reported the photothermal immunoassay of CEA by using polyaniline@AuNPs nanohybrids [240]. Song et al. developed a photothermal biosensor with a temperature and pressure dual-readout for CRP detection based on the in situ generation of polypyrrole [241]. In this work, the detection antibody-modified magnetic Fe_2_O_3_ particles were used in the sandwich immunoassays. After the formation of immunocomplexes, Fe_2_O_3_ particles were decomposed by HCl, and the released Fe^3+^ ions catalyzed the polymerization of pyrrole into polypyrrole. Under constant illustration with an 808 nm NIR laser, the formed polypyrrole caused the increase in both temperature and pressure in the sealed well, which was recorded by a portable thermometer and a pressure meter, respectively.

As a newly emerged semiconductor nanomaterial, conjugated polymer dots show a wide range of optical absorption and unique photostability. By carefully designing the backbone, polymer dots can possess strong NIR absorbance and high photothermal conversion efficiency for phototherapy and biosensing [242]. Wang et al. developed a photoelectrochemical and photothermal immunoassay platform for sensitive detection of sialic acid based on tetraphenylporphyrin-polymer dots (TPP-Pdots) [237]. As illustrated in Figure 21D, rutile-TiO_2_ with excellent photocatalytic activity was used to load TPP-Pdots and antibodies (R–TiO_2_@TPP-Pdots@Ab). The electrode was sequentially decorated with C_60_ and AuNPs, and then 4-mercaptophenylboronic acid (4-MPBA) was assembled on the electrode surface via the Au-S interaction. Sialic acid was captured by the 4-MPBA-modified electrode through the boronate affinity interaction and then labeled with R–TiO_2_@TPP-Pdots@Ab. The e^−^–h^+^ recombination was suppressed by R–TiO_2_@TPP-Pdots, resulting in an increased photoelectrochemical signal. The intensive NIR-absorbing capability and high photothermal effect of TPP-Pdots led to an elevated temperature for photothermal detection.

### 2.8. Other Materials

**Figure 22:**
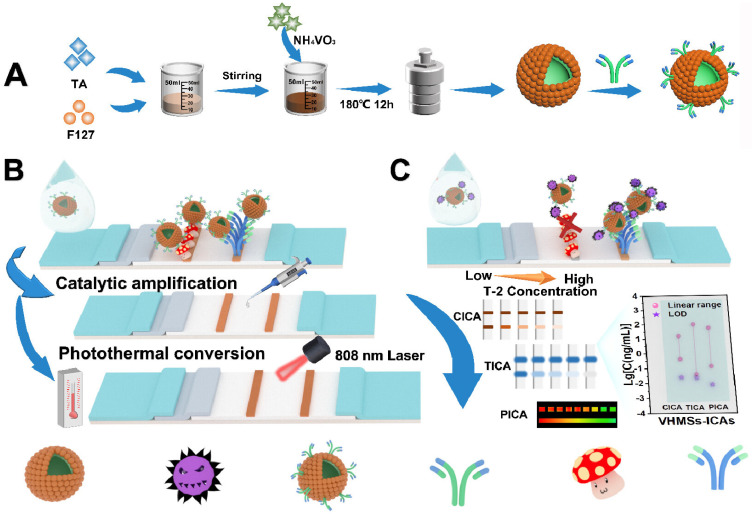
Schematic illustration of a “three-in-one” multifunctional vanadium-based hollow nanocages with colorimetric, photothermal, and catalytic activities for multi-signal immunoassay [244]. Copyright 2024 American Chemical Society.

Vanadium has multiple valence states, which is the basis of vanadium-based nanozymes. It has been documented that vanadium-cross-linked TA exhibited excellent photothermal properties [243]. Recently, Wu et al. prepared a “three-in-one” multifunctional vanadium-based hollow nanocage with colorimetric, photothermal, and catalytic activities for multi-signal immunoassays (Figure 22) [244]. In this work, multifunctional hollow vanadium nanomicrospheres (VHMSs) with a photothermal conversion efficiency of 46.013% were fabricated by formaldehyde-assisted chelating coordination of vanadium ions with TA through a self-templated assembly strategy. The hollow nanocage structures provided more exposed active sites and could accelerate the transfer rate of substrates, improving the catalytic activity of nanozymes. After the competitive immunoassays, vanadium nanomicrospheres catalyzed the oxidation of TMB by H_2_O_2_ to deepen the color of the strip for signal amplification. Meanwhile, the temperature change was recorded under irradiation of an 808 nm laser based on the photothermal effect of vanadium nanomicrospheres.

**Table 7:** Performances of photothermal immunoassays based on polymers and other materials.

Signal Label	Target	Linear Range	Detection Limit	Ref.
AuNPs@PDA	SEB	1–256 ng/mL	0.58 ng/mL	[231]
PCu	A. flavus	1–10^5^ ng/mL	0.22 ng/mL	[232]
Cu_3_(PO_4_)_2_@PDA	CRP	0.42–16 pmol/L	0.11 pmol/L	[234]
CMS@PDA	*S. T*	10^3^–10^7^ CFU/mL	10^3^ CFU/mL	[235]
Fe_3_O_4_@MnO_2_	Exosome	45–4.5 ×10^6^ particles/mL	19.1 particles/mL	[236]
TPP-Pdots	Sialic acid	3.5 × 10^−5^–35 ng/mL	12 fg/mL	[237]
PANi@Au	CEA	0.2–25 ng/mL	0.17 ng/mL	[240]
Fe_2_O_3_	CRP	0.75–12 mg/L	0.45 mg/L	[241]
VHMS	T-2 toxin	0.122–100 ng/mL	2 pg/mL	[244]

Abbreviations: AuNPs@PDA, polydopamine-coated gold nanoparticles; SEB, staphylococcal enterotoxin B; PCu, Cu-anchored photothermal polydopamine; *A. flavus*, *Aspergillus flavus*; Cu_3_(PO_4_)_2_@PDA, polydopamine-coated or encapsulating Cu_3_(PO_4_)_2_; CRP, C-reactive protein; CMS@PDA, two-dimensional nanomaterial (Cu_2_MoS_4_)-modified polydopamine; *S. T*, *S. typhimurium*; TPP-Pdots, tetraphenylporphyrin-polymer dots; PANi@Au, polyaniline@Au organic- inorganic nanohybrid; CEA, carcinoembryonic antigen; VHMS, hollow vanadium nanomicrosphere.

## 3. Conclusions

In this review, photothermal immunoassays based on different types of photothermal nanomaterials were summarized. Compared with other methods, photothermal immunoassays show outstanding advantages, including direct signal readout, controllable sensing, and low background interference. Although many achievements have been made in recent years, there are still some key challenges that need to be addressed to further stimulate the development of photothermal immunoassays with improved analytical performance. First, the design and preparation of novel and multifunctional photothermal agents play a key role in the development of such immunoassays. Tailoring the parameters of photothermal nanomaterials (e.g., size, shape, and composition) can achieve high photo-to-heat conversion efficiency. Second, complex environments with high background noise and various competing targets are challenges to achieving accurate immunoassays, and the result may be affected by sunlight or artificial light, ambient temperature and humidity, and other physical vibrations. Thus, the environmental stability of photothermal immunoassays needs to be addressed, and some highly environmentally stable nanocomposites, chemical coatings, optical filtration devices, and physical packages can be developed to improve detection accuracy. Third, although photothermal immunoassays show simplicity, convenience, and excellent portability, their sensitivity and detection limit still need to be improved. Effective signal amplification strategies should be developed to enhance the photothermal detection sensitivity and decrease the detection limit. Fourth, a couple of antibodies used in immunoassays are always high cost and require strict storage and detection conditions. Much effort should be devoted to developing effective and stable artificial/synthetic receptors in place of the capture or recognition antibody in sandwich immunoassays. Finally, the device for photothermal signal readout plays a crucial role in photothermal detection, which is closely associated with the analytical performance of immunoassays. Reaction vessels with thermal insulation properties and appropriate volumes may significantly reduce the loss of heat and guarantee the accuracy and sensitivity of photothermal immunoassays. Thus, more efforts should be made in developing effective miniaturized and integrated temperature readers and POCT devices (e.g., microfluidics by 3D printing or lithography) for high throughput, sensitive, and real-time analysis.

## Data Availability

Data are contained within the article.
